# Lentinan alleviates arsenic-induced hepatotoxicity in mice via downregulation of OX40/IL-17A and activation of Nrf2 signaling

**DOI:** 10.1186/s40360-022-00557-7

**Published:** 2022-03-22

**Authors:** Yuan Yang, Shuang Song, Yuanyuan Nie, Rong Chen, Peng Chen

**Affiliations:** 1 School of Public Health, The Key Laboratory of Environmental Pollution Monitoring and Disease Control, Ministry of Education, Guizhou Medical University, Guiyang, 550025 China; 2 Dong Medicine Key laboratory of Hunan Province, Department of Laboratory Medicine, Hunan University of Medicine, Huaihua, 418000 China; 3 Department of Toxicology, School of Public Health, Guilin Medical University, Guilin, 541004 China

**Keywords:** Arsenic, Hepatotoxicity, Lentinan, Inflammation, Antioxidation

## Abstract

**Background:**

Arsenic, existing ubiquitously in soil, drinking water, or food, is well known to be an environmental pollutants concerned by European Food Safety Authority. *Lentinan,* a beta-1,6;1,3-glucan extracts from *Lentinus edodes*, which has the properties of antioxidant and immunomodulation, present study explored the pharmacological effects of *Lentinan* on arsenic induced hepatotoxicity in mice.

**Methods:**

Mice experiments were performed by sodium arsenite (SA) treatment or *Lentinan* intervention, then histopathology, ELISA, Flow Cytometry, or Western-Blotting were applied to evaluate hepatic injury, oxidative stress, CD4^+^ type 17 helper T (Th17) cells, CD4^+^CD25^+^Foxp3^+^ regulatory T cells (Tregs), T cells receptor OX40/CD134, IL-17A, NLRP3, Nrf2, and NQO1.

**Results:**

SA treatment showed hepatic pathological injury and the elevations of alanine aminotransferase (ALT) or aspartate aminotransferase (AST) in serum, and induced the increases of malondialdehyde (MDA), Th17 cells, OX40 or IL-17A in liver tissues, which were consistently ameliorated by *Lentinan* intervention. Further, immunoblotting experiments showed that *Lentinan* intervention downregulated the levels of OX40, IL-17A, and NLRP3 signals, while elevated the levels of anti-oxidative Nrf2, NQO1 signals compared to arsenic treatment group. For Tregs, *Lentinan* intervention showed no significant difference from SA treatment group.

**Conclusion:**

*Lentinan* antagonizes SA-induced hepatotoxicity in mice, may be involved in the downregulations of pro-inflammatory OX40 or IL-17A and the activation of anti-oxidative Nrf2, NQO1 signals.

**Supplementary Information:**

The online version contains supplementary material available at 10.1186/s40360-022-00557-7.

## Introduction

Arsenic, existing ubiquitously in environment, which causes the increased risk of lung, skin and bladder cancer. Particularly, the ‘milk and dairy products’, ‘drinking water’ and ‘food for infants’ and rice is an important source of inorganic arsenic, concerned by European Food Safety Authority (EFSA) [1, 2]. Regarding the arsenic-induced hepatic toxicity, the underlying mechanism demonstrated as the imbalance of oxidant-antioxidant, the elevations of pro-inflammatory cytokines, DNA damage, apoptotic cell death, and pyroptosis [3, 4]. Moreover, our study show arsenic induced the increased levels of proinflammatory cytokines IL-1β, IL-6, and NLRP3 inflammasome in mouse liver [5]. The liver is a frontline immune tissue, and liver function is associated closely with immune response, excessive inflammation in the absence of infection leads to sterile liver injury and tissue damage [6]. Further, recent study demonstrated that arsenic interfered with the differentiation of helper T cells (Th) such as Th1/Th2/Th17 subsets in lung and spleen of mice [7]. Mechanistically, nuclear factor erythroid 2 related factor (Nrf2), a master transcription factor, its activation can suppress Th17 immune responses and rectify oxidant-antioxidant imbalance in periphery and brain of BTBR T + tf/J (BTBR) mice [8]. And, the activated Nrf2 can downregulate oxidative stress and inflammation parameters (NF-κB, IL-6, IL-1β) in autistic children, which is responsible for the ameliorative effects in Autism spectrum disorder (ASD) subjects [9], indicating Nrf2 play a significant role by its management of anti-inflammatory and antioxidant genes in arsenic-induced hepatotoxicity.

*Lentinan* (LNT) is the bioactive polysaccharide of beta-1,6;1,3-glucan, isolated from the mushroom shiitake *Lentinus edodes* (L. edodes), which has the high capability of antioxidation against cellular oxidative damage and preserving cell activity [10], and showing the anti-inflammatory activity of inhibiting inflammatory cytokine production [11]. For example, LNT alleviates against cisplatin-induced nephrotoxicity by the inhibitions of reactive oxygen species (ROS) and the activation of the Nrf2-antioxidant response element (ARE) signalling pathway in kidney [12]; LNT attenuates IL-1β secretion resulting from Listeria-mediated AIM2 inflammasome activation in myeloid cells [13]. Interestingly, the polysaccharides in Lentinus edodes possess the immunomodulatory activity of activating different immune responses in the host [14]. LNT cancels Th2-dominant condition and decreases significantly the percentages of CD4^+^IL-4^+^ T-cell and CD4^+^ IL-6^+^ T-cell in patients with digestive cancers [15], and LNT inhibits the activation of Th2 cells and affects the immune microenvironment, leading to the alleviated allergic reactions in allergic mice [16]. These studies indicate LNT’ bioactivity is cross-linked to arsenic toxicity under the pathological status of poisoning and injury, so the present study investigated the pharmacological effects of LNT by a mice model of sodium arsenite (SA) oral administration.

## Materials and methods

### Animal experimental protocol

The methodology of the present study was reviewed, and approved by Ethics Committee of Guizhou Medical University (no. 1900222). Male C57BL/6 mice weighed 25.5 ± 1.0 g, age range from 9 to 10 weeks, were obtained from the Slack King of Laboratory Animal Co., Ltd. (certificate of conformity: SCXK2019–0004, Changsha, China). Thirty-two mice were randomly allocated into four groups (*n* = 8/group). (i) Control group: mice mice were administrated orally by deionized water (0.25 ml/time, once every other day for 14 days); (ii) SA treatment group: mice were administrated by gavage sodium arsenite (NaAsO_2_) (CAS.7784-46-5, Sigma-Aldrich, USA) solution (dissolved in deionized water by 1.0 mg/ml, dose: 10.0 mg/kg.bw, 0.25 ml/time), once every other day for 14 days, referred to Bashir et al. methods [3, 17]; (iii) LNT treatment group: mice were administered by intramuscular injection LNT (CAS.37339–90-5, Shanghai Duma Biotechnology Co., Ltd.; dissolved in deionized water, dose: 1.0 mg/kg.bw), once every other day for 14 days, referred to Mao et al. studies [18, 19]; (iv) LNT + SA treatment group: mice were treated by LNT plus SA, LNT was administered 8 h ahead of the SA treatment, referred to the methods of (ii) and (iii). When the experiment was complete, the mice were euthanized by intraperitoneal injection of sodium pentobarbital, then blood samples were collected and liver tissues were immediately isolated, one part of liver tissue from each mouse was fixed in 4% phosphate-buffered formaldehyde for hematoxylin-eosin (HE) staining, and the rest of liver tissue was placed in liquid nitrogen for further analysis.

### Liver function, HE staining for histopathological evaluation

Blood samples were centrifuged at 4 °C and 400×*g* for 5 min, and the serum supernatants were collected for measuring the levels of ALT (MLGR-E21533), AST (MLGR-E21534) according to the instructions of ELISA assay kit (Shanghai Mlbio Biotechnology Co., Ltd.). For the evaluation of pathological liver injury, liver tissues were fixed with formaldehyde for 72 h, and then embedded in paraffin for preparing 5 μm pathological sections. HE staining was performed for evaluating the histopathological characteristics of liver tissue sections under Motic microscope (DMB5-2231P1).

### Measurement of Th17, Tregs in liver tissues by flow Cytometry

Determination of Th17, Tregs referred to Kim MH, et al. method [20]. Briefly, liver tissues were homogenated to collect the constituent cells, and were washed twice with phosphate buffer saline (PBS) for obtaining cell suspension, then were added Cell Stimulation Cocktail 500 × (00–4975-93, eBioscience), at 37 °C incubator for 6 h, and the collected cells were treated by Fixation/Permeabilization Concentrate 4*×* under 4 °C for 30 min. Cells were re-suspended, and the cells suspensions were incubated with anti-CD4 (11–0041-82, eBioscience) and anti-IL-17 (45–7177-82, eBioscience) antibodies at 4 °C for 30 min for evaluating the content of Th17 cells. Alternatively, the cells suspensions were incubated with the anti-CD4 (11–0041-82, eBioscience), anti-CD25 (17–0251-82, eBioscience) and anti-Foxp3 (12–5773-82) antibodies (ebioscience, USA) at 4 °C for 30 min for evaluating the content of Tregs, according to standard procedures. After two washes with binding buffer, the collected cells were analyzed using the AQUIOS CL Flow Cytometry System (Beckman Coulter, Inc., San Jose, Brea). For gating controls in flow cytometry, FcR Blocking Reagent (130–092-575, Miltenyi) and the isotype control for each antibody, were used during flow cytometry, respectively.

### Detection of oxidative stress indices in liver tissues

Briefly, liver tissues were dissected out, and were rinsed with PBS to remove excess blood. Then tissues were homogenized in PBS (10% w/v) by Dounce Homogenizer (BioVision) and were incubated with RPMI media containing 0.05% collagenase type II (C2-BIOC, Sigma-Aldrich) at 37 °C for 30 min. Then the homogenates were done by three repeated freeze-thaw cycles of lysing cells, subsequently centrifuged at 4 °C, 1500×*g* for 10 min, the final supernatants were collected for detecting the content of MDA (MLGR-E20206, Shanghai Mlbio Biotechnology Co., Ltd.) or Glutathione (GSH) (MLGR-E20324, Shanghai Mlbio Biotechnology Co., Ltd.) by ELISA method. The OD values were measured by Microplate reader (BioTek800 TS, USA) at wavelength of 450 nm, and the measured results were converted from OD values to the nanomolar concentration (nmol/g wet tissue) according to the standard curve and dilution ratio.

### Measurement of OX40, IL-17A in liver mononuclear cells

Liver mononuclear cells (LMC) referred to Zhang J et al. methods of digestion and filteration [21]. Briefly, the dissociated liver tissues were homogenized and digested by 0.05% collagenase type II for 30 min at 37 °C. The cells mixture was then filtered through a 70-μm nylon cell strainer and centrifuged at 500×*g* for 5 min. Further, the supernatants were collected for quantitative analysis of T cells membrane receptor OX40/CD134 (EK0998, Wuhan Boster Biotechnology Co., Ltd.) or inflmmatory cytokine IL-17A (MLGR-E20171, Shanghai Mlbio Biotechnology Co., Ltd.) by ELISA method according to the manufacturer’s instructions, respectively.

### Western-blotting evaluation of inflammatory or anti-oxidative signaling

Following liver tissues were routinely dissociated and digested by collagenase type II, the hepatic cells suspensions were collected for extracting total proteins by Total Protein Extraction Kit (Shanghai Sangon Biotech, China) according to the manufacturer’s instructions, then the concentration of proteins was determined using the Bradford reagent (Sigma-Aldrich, USA). The expressions of OX40, Nrf2, NQO1, and inflammatory signaling NOD-like receptor family pyrin domain-containing 3 (NLRP3) were evaluated by the standard steps of Western-blotting (WB) experiment. The primary antibodies were provided as the followings: Anti-CD134/OX40L receptor antibody (ab229021, 1:800, Abcam), anti-Nrf2 antibody (ab92946, 1:1000, Abcam), anti-NQO1 antibody (sc-32,793, 1:800, Santa Cruz Biotechnology), or anti-NLRP3 antibody (ab263899, 1:1000, Abcam). Anti-rabbit IgG-HRP antibody (LS-C86382, LSBio) was used as the secondary antibody. WB results were pictured by enhanced chemiluminescence reagent (Beyotime, China), and then the integrated absorbance (IA) of the bands were analyzed quantitatively using LI-COR Odyssey Imaging System. Anti-GAPDH Rabbit Polyclonal antibody (BM1623, Dingguo Biotech Co. Ltd) were used as an internal control for normalizing the values in equal samples, the relative levels of target proteins were evaluated by the ratio of target protein vs internal control (IA/IA).

### Statistical analysis

Data were expressed as mean ± standard deviation (SD), and were analyzed by *SPSS* version 23.0 (IBM Corp). One-way analysis of variance (ANOVA) test and independent sample’s t-test were applied to evaluate the statistical difference between groups. *P*<0.05 was considered as a significant difference between groups.

## Results

### LNT intervention alleviated hepatic damage induced by arsenic

**Fig. 1:**
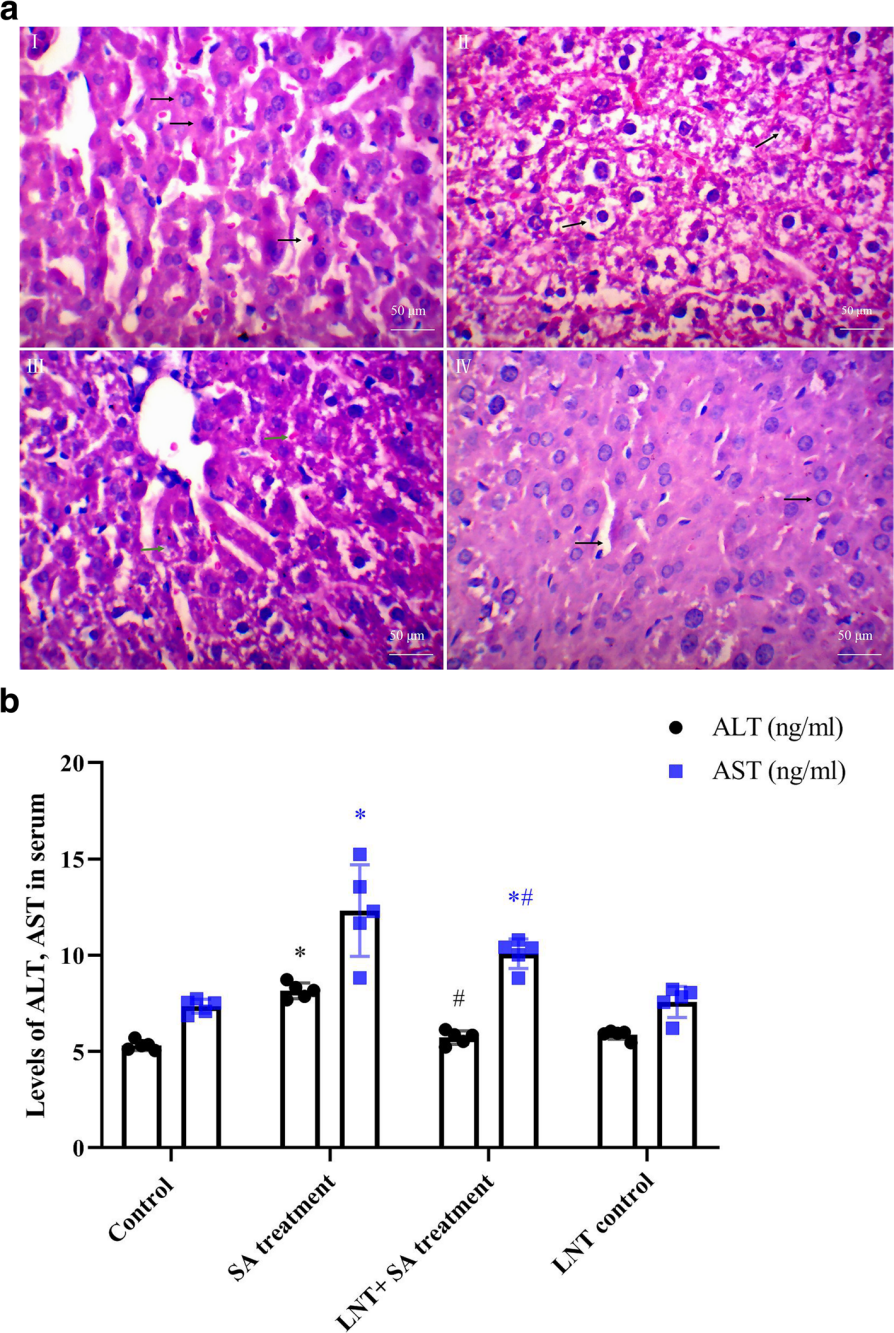
Characterisrics of hepatic histopathology or liver function in mice. **A** Representative histopathological images (HE, × 200) in liver. (I) Control: Liver histology was normal, as shown by the arrow, liver cell nucleus uniform, the nuclear membrane smooth, the chromatin stained lightly, the cytoplasm uniformly pink, hepatocyte cord visible, and hepatic sinus no dilation and arrangement regular; (II) SA treatment group: Mice were administrated orally by SA at 10.0 mg/kg.bw once every other day for 14 days, as shown by the arrow, hepatocyte degeneration or even disappear, hepatocyte edema and vacuolar degeneration, accompanied by the disconnected hepatocyte cord and the unclear boundaries of hepatocytes; also, some places showd the different sizes of nucleus and the nucleus shrinkage or even disappear; (III) LNT + SA treatment: Mice were injected intramuscular with LNT at 1.0 mg/kg.bw and subsequent oral administration of SA at 10.0 mg/kg.bw once every other day for 14 days, as shown by the arrow, hepatocyte cord was visible, hepatic sinusoids expansion mild, less severe than SA treatment group, hepatocytes shoed mild to moderate edema, the sizes of dense staining nuclear varied, and nuclear membrane mild thicken; (IV) LNT control: The normal histology of liver tissues were similar to control group, as shown by the arrow, hepatocyte showed normal without edema and the normal ratio of nuclear to plasma, the uniform nucleus size, the smooth nuclear membrane, the lightly stained chromatin, hepatic sinusoids showed normal shape and no dilatation. **B** Histogram of ALT or AST content in serum after SA treatment or *Lentinan* intervention. SA treatment showed the increased levels of serum ALT or AST, which was downregulated significantly after the intervention with *Lentinan*; Data were expressed as mean ± SD; *n* = 5. Levels of ALT, AST in serum were quantified by the the convertion from OD values to ng values of per milliliter according to the standard curve; Independent sample’s t-test followed ANOVA was performed for analyzing the difference between groups. *P < 0.05 compared with control group; ^#^*P* < 0.05 compared with SA treatment group. Abbreviations: SA, sodium arsenite; HE, hematoxylin-eosin; LNT, Lentinan; ALT, alanine aminotransferase; AST, aspartate aminotransferase

As shown in Fig. 1A and B, HE staining sections showed hepatic injury in SA treatment group, such as hepatocytes edema, degeneration, nucleus shrinkage, and even some hepatocytes disappear, hepatocyte cord disconnection, and hepatocytes boundaries unclear. Interestingly, the intervention with LNT showed an amelioration of liver pathological injury, characterized as the alleviated hepatocytes edema, the decreased expansion of hepatic sinusoids compared with that in SA treatment group. LNT intervention showed also the significant antagonism against the high levels of ALT or AST in SA treatment group (ALT: *F* value as 0.82, t value as 6.43, *P* < 0.05; AST: *F* value as 4.61, t value as 2.85, *P* < 0.05). The results suggest the improvement of liver dysfunction and a beneficial role of LNT intervention, details were shown as Table S1 (Supplementary material).

### LNT intervention downregulated Th17 levels in liver against arsenic treatment

**Fig. 2:**
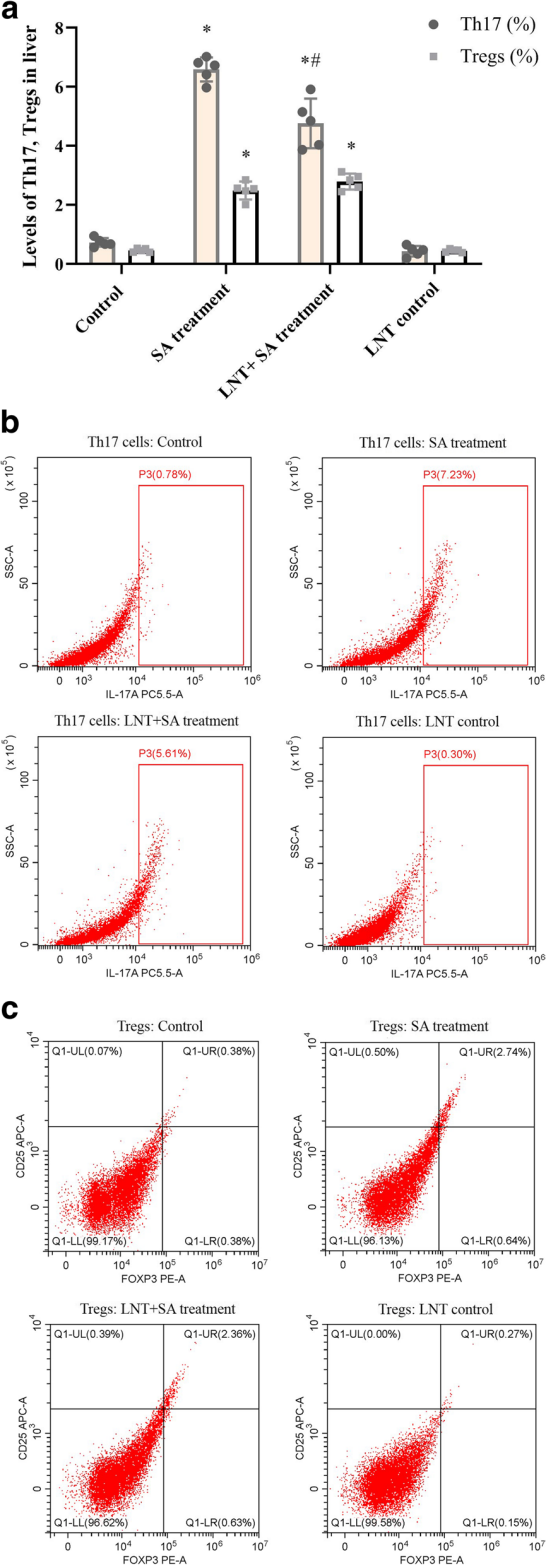
Characteristics of Th17 or Tregs in liver after arsenic exposure or *Lentinan* intervention. **A** Characteristics of Th17 or Tregs in liver tissues after SA exposure or *Lentinan* intervention. SA treatment showed the upregulated levels of Th17 or Tregs in liver tissues compared to control, and the intervention *with Lentinan* led to a significant downregulation of Th17 compared to SA treatment group (*P* < 0.05), while the change of Tregs was no significant (*P* > 0.05); **B** Representative images of Th17 in liver tissues by Flow cytometry analysis; **C** Representative images of Tregs in liver tissues by Flow cytometry analysis. Data were showed as mean ± SD; *n* = 5. Th17, Tregs were presented as percentage (%), which was analyzed by ANOVA and independent sample’s t-test; ^*^P < 0.05 indicates a significant difference compared with control group; ^#^*P* < 0.05 indicates a significant difference compared with SA treatment group. Abbreviations: Th17, CD4^+^ type 17 helper T cells; Tregs, CD4^+^CD25^+^Foxp3^+^ regulatory T cells

As shown in Fig. 2A, B, C, SA treatment showed a significant increase of Th17 lymphocytes in liver tissues compared to control group (*F* value as 3.47, t value as 31.32, *P* < 0.05). And, SA induced a significant downregulation of Th17 lymphocytes in liver tissues was revealed after LNT intervention compared with that of SA treatment group (*F* value as 0.04, t value as 9.41, *P* < 0.05). For the Tregs, SA treatment showed the elevated level of Tregs in liver tissues compared to control group (*F* value as 4.28, t value as 6.80, *P* < 0.05), while the intervention with LNT showed no significant difference from SA treatment group (*F* value as 1.99, t value as 0.79, *P* > 0.05), details were shown as Table S2 (Supplemental material).

### LNT downregulated MDA and inflammatory signals during arsenic toxicity

**Fig. 3:**
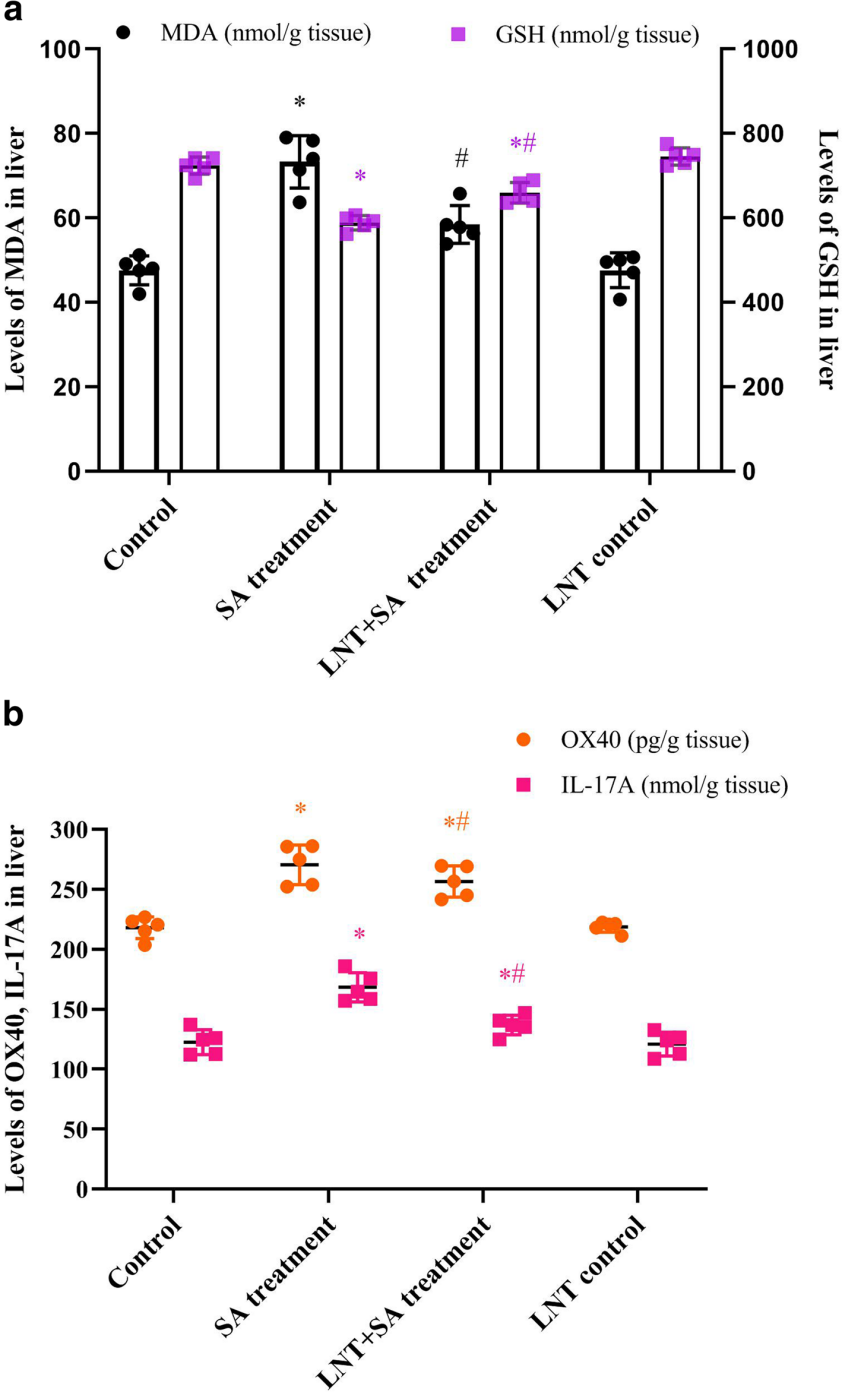
*Lentinan* antagonized oxidative stress and inflammatory signals in liver. **A**. Histogram of oxidative stress indicators MDA, GSH after SA treatment or *Lentinan* intervention. An increased MDA and a reduced GSH in liver were found in SA treatment group, which were antagonized consistently by *Lentinan* intervention; **B** Characteristics of inflammatory signals OX40, IL-17A after SA treatment or *Lentinan* intervention. SA induced the upregulations of proinflammatory signals OX40, IL-17A, while was downregulated significantly after *Lentinan* intervention. Data were expressed as mean ± SD; *n* = 5. The levels of MDA, GSH, OX40, and IL-17A in liver tissues were quantified by the convertion of OD values to nanomole or picogram values of per gram liver tissue (nmol or pg/g tissue) according to the standard curve and dilution ratio. Significant difference was obtained by independent sample’s t-test followed ANOVA. ^*^P < 0.05 indicates a significant difference compared with control; ^#^*P* < 0.05 indicates a significant difference compared with SA treatment. Abbreviations: IL, Interleukin; MDA, malondialdehyde; GSH, glutathione; OX40 (CD134), Tumor necrosis factor receptor superfamily member 4

As shown in Fig. 3A, compared to control group, SA treatment group showed an increase of oxidative stress index MDA and a reduce of antioxidant GSH in liver tissues (MDA: *F* value as 0.38, t value as 3.80, *P* < 0.05; GSH: *F* value as 0.27, t value as 11.17, *P* < 0.05), indicating the occurrence of hepatic oxidative damage after SA exposure. After the pretreatment intervention with LNT, a significant downregulation in MDA and the rebound of GSH were found as a comparison with SA treatment group (MDA: *F* value as 0.05, t value as 2.50, *P* < 0.05; GSH: *F* value as 0.47, t value as 3.53, *P* < 0.05), suggesting an alleviation of hepatic oxidative stress elicited by LNT intervention.

As shown in Fig. 3B, compared to control group, a significant increase of T lymphocytes immunity associated inflammatory signals OX40, IL-17A in liver were revealed in SA treatment group (OX40: *F* value as 1.17, t value as 7.60, *P* < 0.05; IL-17A: *F* value as 0.38, t value as 7.76, *P* < 0.05). Compared to SA treatment group, the intervention with LNT showed a significant downregulation against the high levels of OX40, IL-17A (OX40: *F* value as 0.11, t value as 2.76, *P* < 0.05; IL-17A: *F* value as 0.13, t value as 6.09, *P* < 0.05). The results indicate an amelioration of CD4^+^ or CD8^+^ T cell mediated inflammation in liver, more details were shown in Table S3 (Supplemental material).

### LNT inhibited inflammatory signaling and activated Nrf2 in liver

**Fig. 4:**
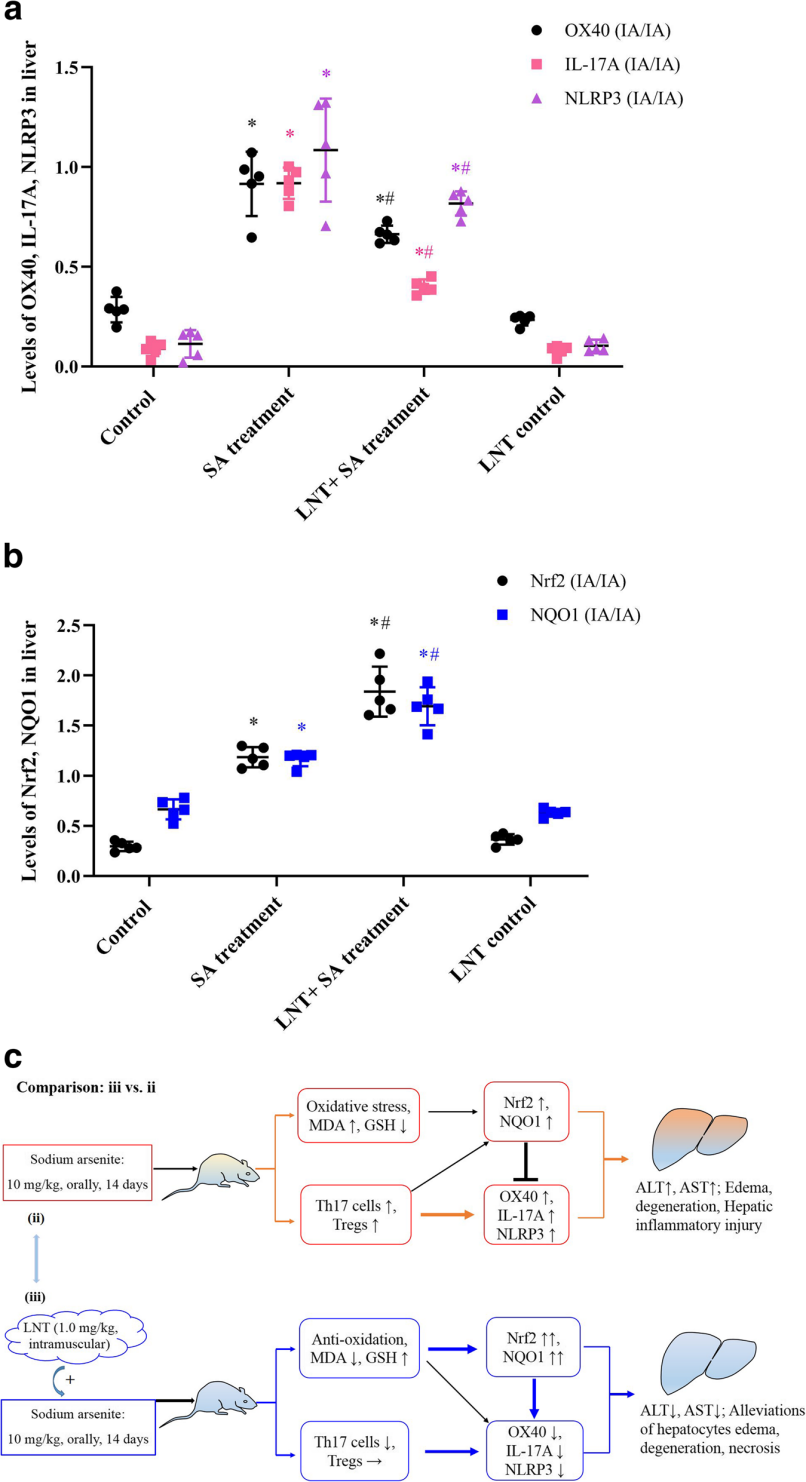
*Lentinan* downregulated arsenic-induced inflammatory signals and upregulated anti-oxidative signals in liver by western-blotting analysis. **A** Characteristics of OX40, IL-17A, and NLRP3 inflammatory signaling after SA exposure or *Lentinan* intervention. SA induced a significant upregulations of OX40, IL-17A, and NLRP3, which were antagonized significantly after the intervention with *Lentinan* (*P* < 0.05); **B** Characteristics of Nrf2, NQO1 after SA exposure or *Lentinan* intervention. SA induced the upregulations of Nrf2 and NQO1, while the intervention with *Lentinan* showed the further upregulations of Nrf2 and NQO1 compared to SA treatment group (*P* < 0.05). Data were expressed as mean ± SD; *n* = 5. The levels of proteins expression were evaluated by the measurement of relative IA levels, quantified by the ratio of detected protein vs. internal control (β-actin) in each group (IA/IA). The significant difference between groups was obtained by independent sample’s t-test followed ANOVA. ^*^P < 0.05 indicates a significant difference compared with control group; ^#^*P* < 0.05 indicates a significant difference compared with SA treatment group. Abbreviations: NLRP3, NOD-like receptor family pyrin domain-containing 3; Nrf2, NF-E2 p45-related factor 2; NQO1, NAD(P)H quinone dehydrogenase 1; IA, integrated absorbance

As shown in Fig. 4A, the expressions of OX40, IL-17A and NLRP3 inflammatory signals showed a significant upregulation in SA treatment group as compared to control group (OX40: *F* value as 0.46, t value as 15.58, *P* < 0.05; IL-17A: *F* value as 0.86, t value as 54.26, *P* < 0.05; NLRP3: *F* value as 11.39, t value as 7.92, *P* < 0.05). While the intervention with LNT showed a significant downregulation in OX40, IL-17A and NLRP3 signals compared to SA treatment group (OX40: *F* value as 6.16, t value as 7.68, *P* < 0.05; IL-17A: *F* value as 0.64, t value as 18.13, *P* < 0.05; NLRP3: *F* value as 0.27, t value as 6.49, *P* < 0.05). Therefore, the WB experiments further demonstrated the intervention with LNT led to an amelioration against the immunity-related hepatic inflammation induced by SA,

As shown in Fig. 4B, compared to control group, the high levels of anti-oxidative Nrf2 and its downstream target NQO1 signaling were revealed in SA treatment group (Nrf2: *F* value as 1.42, t value as 14.31, *P* < 0.05; NQO1: *F* value as 1.40, t value as 4.18, *P* < 0.05). While the intervention with LNT showed the further upregulations of Nrf2, NQO1 compared to SA treatment group (Nrf2: *F* value as 1.98, t value as 7.21, *P* < 0.05; NQO1: *F* value as 0.02, t value as 4.29, *P* < 0.05). The results indicate the activation Nrf2-ARE signaling pathway maybe as an intracellular stress response during arsenic toxicity, while the further activation of Nrf2-ARE signaling pathway indicated the enhanced hepatic anti-oxidative capability after LNT intervention, details were shown as Fig. S1 ~ 2 & Table S4 (Supplemental material).

## Discussion

Th17 cells, as a pro-inflammatory type of T Lymphocyte subtypes originated from the differentiation of precursor T helper cell 0 (Th0), which were developed from naive CD4^+^ T cell after antigen stimulation. Th17 differentiation can be directed by their master transcription factor retinoic acid receptor-related orphan receptor-γt (RORγt). RORγt as a specific transcript of the RORC gene regulated by transcription factors signal transducer and activator of transcription 3 (STAT3), are involved in mediating inflammatory responses and exacerbating liver injury by inducing the release of pro-inflammatory cytokines, such as IL-17 and tumor necrosis factor-alpha (TNF-α) [22]. IL-17 as the proinflammatory cytokine secreted by Th17 cells, IL-17 binds to its receptor, can induces the activation of NF-κB signaling [23], and the activated NF-κB signaling further up-regulates the NLRP3 inflammasome response [24], while blocking NLRP3 inflammasome reduces liver inflammation and fibrosis in experimental liver disease model in mice [25]. In the present study, arsenic exposure led to hepatic pathological injury and oxidative stress, accompanied by the elevated proportion of Th17 cells, and the upregulations of inflammatory signals IL-17A, NLRP3 in mouse liver. The results indicate that arsenic exposure activated transcription factors RORγt, STAT3. Consequently, the hepatic augment of Th17 cells induced inflammatory signal IL-17A production, and then IL-17A mediated the activation of NF-κB inflammatory signaling pathway, which is responsible for the pathological injury in liver. Interestingly, the intervention with LNT showed an effective antagonism against arsenic-induced the elevations of IL-17A and inflammasomes NLRP3 in mice, indicating the downregulation in IL-17A secreted by Th17 cells, contributing to the amelioration of downstream NF-κB inflammatory signaling in hepatocytes and the attenuation of arsenic-induced hepatotoxicity in mice. Similarly, the previous studies showed that LNT reduces the activation of NF-κB and inhibits the production of pro-inflammatory cytokines IL-1β, TNF-α, IL-8 in chondrocytes [26], LNT intervention improves hepatic cell morphology by the decreased activity of NF-κB signal in rat’s liver with sepsis [27].

Also, the present study showed that LNT intervention leads to the antagonism against arsenic-induced oxidative stress in liver of mice. Oxidative stress as an intracellular pathological state, indicating the reduced antioxidants and the increased ROS in cells, which is considered as the common pathophysiological characteristic during arsenic induced multiple organ injury such as liver, kidney, skin, etc. [28]. Mechanistically, oxidative stress can elicit DNA damage, leads to mitochondrias dysregulation, endoplasmic reticulum stress, and chronic inflammation [29]. Consequently, the inhibition of oxidative stress leads to the upregulations of anti-oxidative Nrf2 signaling pathway [30], and leads to the amelioration of hepatic inflammation or acute liver injury, involved in the downregulations of NF-κB inflammatory signaling [31]. Nadeem A et al. demonstrated that the activated Nrf2 signal antagonizes NF-κB signaling and inhibits inflammatory parameters [9]. Here, despite the activated Nrf2 signaling induced by arsenic exposure in the present study, the intervention with LNT showed that the amplified elevation of Nrf2-ARE signaling pathway. It is speculated that arsenic induced the activations of Nrf2 and its downstream signaling in mice, which is considered as an intracellular stress response in body, as evidenced by Liu D et al’ study that inorganic arsenic induces the activation of the Nrf2 pathway in Chang human hepatocytes [32]. Significantly, LNT intervention induced the amplified elevation of activated Nrf2, NQO1 signals, indicating the effective inhibitions of oxidative stress, inflammatory cytokines production in hepatocyte, which maybe is involved in its immunomodulation bioactivity of T lymphocytes immune related inflammatory cytokines in arsenic-induced hepatic injury. Indeed, the previous studies demonstrated that LNT can suppress Th17 immune responses in BTBR mice [8], and demonstrated that the antioxidant Nrf2 signaling pathway is associated negatively with the regulation of liver inflammation [33]. Mechanistically, the activated Nrf2 promotes itself nuclear translocation, which, in turn, up-regulated phase II/antioxidant enzyme activities, and simultaneously reduces inflammatory response originated from hepatic injury [34]. For example, the Nrf2 activates its anti-oxidative downstream heme oxygenase-1 (HO-1) signaling pathways, which contributes to the amelioration of hepatic inflammation cell infiltration [35].

Interestingly, the downregulation of Th17 cells-associated inflammatory signals OX40 was found after the intervention with LNT. OX40, named as CD134 or tumor necrosis factor-receptor superfamily member 4 (TNFRSF4), a member of tumor necrosis factor receptor (TNFR) superfamily expressed on activated T cells, which is defined as a biomarker of T cell activation, whose ligand OX40L is found on activated T and B cells [36]. The bounding of OX40-OX40L play a critical role of regulating the activity of T cell and inflammatory cytokines production, associated closely with the proliferation, differentiation, or activation of CD4^+^ or CD8^+^ T cells [37, 38]. Recent study found that OX40 expressed in innate immune cells such as neutrophils, contributing to the prolonged neutrophils survival, and promoted proinflammatory cytokines, ROS production, while Ox40 knockout markedly alleviated liver injury [39]. In the present study, the intervention with LNT downregulated significantly the levels of OX40, indicating the decreases of innate immune cells and adaptive immune helper T cells, contributing to the amelioration of inflammation in liver. And, the immunomodulating activity of downregulating CD4(+) lymphocyte subsets-associated cytokines such as TNF-α, IL-2, IL-17 production in mice, which was demonstrated by other organic compounds such as β-1,3-Glucan (BG) with similar structure to LNT [40].

Summarily, the present study revealed that arsenic induced the activation of T cell immunity associated inflammatory signaling in mouse liver, while the intervention with LNT showed a hepatoprotective effects, manifested as the significant downregulations of OX40, IL-17A, and the enlarged elevation of Nrf2-NQO1 signaling, outlined as Fig. 4C. In future, our study should aim to explore the potential mechanism between Th1/2 cell differentiation and Nrf2-AGE signaling pathway by the LNT experiments in vitro, expecting to elucidate the cross-link pathway of LNT-regulated intracellular antioxidative Nrf2 signaling pathway against T lymphocytes-associated inflammatory response during arsenic-induced hepatotoxicity.

## Conclusion

Arsenic induces hepatic pathological injury in mouse, while are antagonized significantly by LNT intervention, may be involved in the inhibition of T cell immunity-associated IL-17A or OX40 inflammatory signaling, and the activation of anti-oxidative Nrf2 signaling.

## Supplementary Information

**Additional file 1.**

## Data Availability

All data needed to support the conclusions are included in this article, supplementary data are present in the supplemental materials. Additional data related to this paper can be requested from the author (yang1977yuan@sina.com).
