# Supplementary material for: Lentinan alleviates arsenic-induced hepatotoxicity in mice via downregulation of OX40/IL-17A and activation of Nrf2 signaling
Source: BMC Pharmacol Toxicol. 2022 Mar 22;23:16. doi: 10.1186/s40360-022-00557-7 (PMC8939159; doi:10.1186/s40360-022-00557-7)
Supplement: Supplementary file 1 — Additional file 1. [file 40360_2022_557_MOESM1_ESM.zip › R1_Supplemental material_2022.pdf]

Supplemental Figures

Figure S1. Representative western-blotting images of OX40, IL-17A, and NLRP3 in liver after arsenic exposure or *Lentinan* intervention

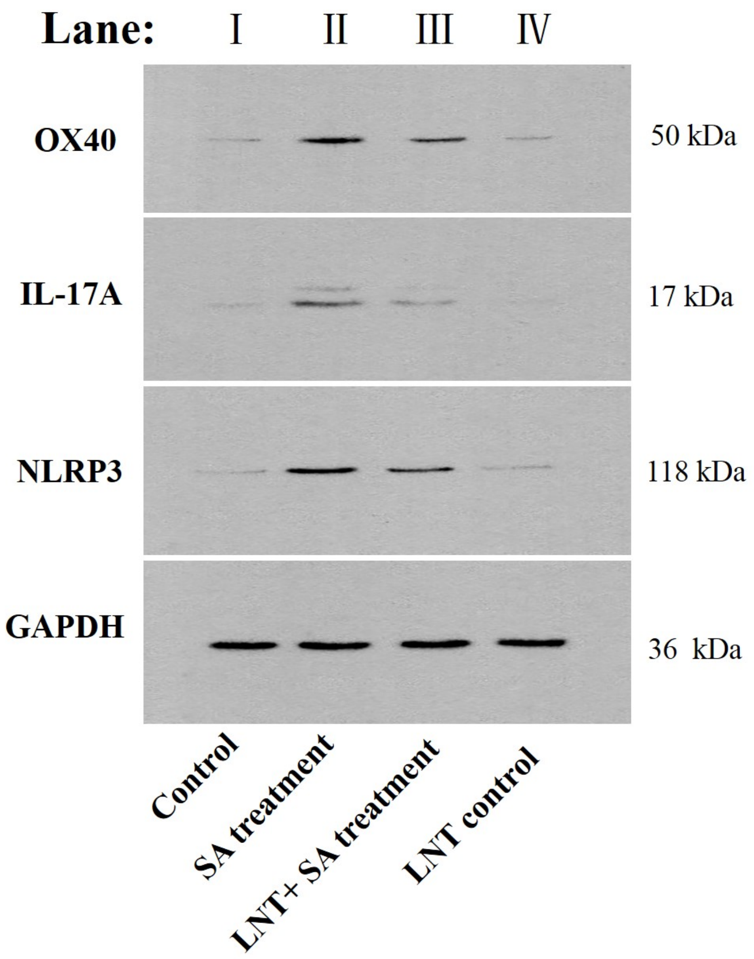

The original figure S1: Each figure has two exposures.

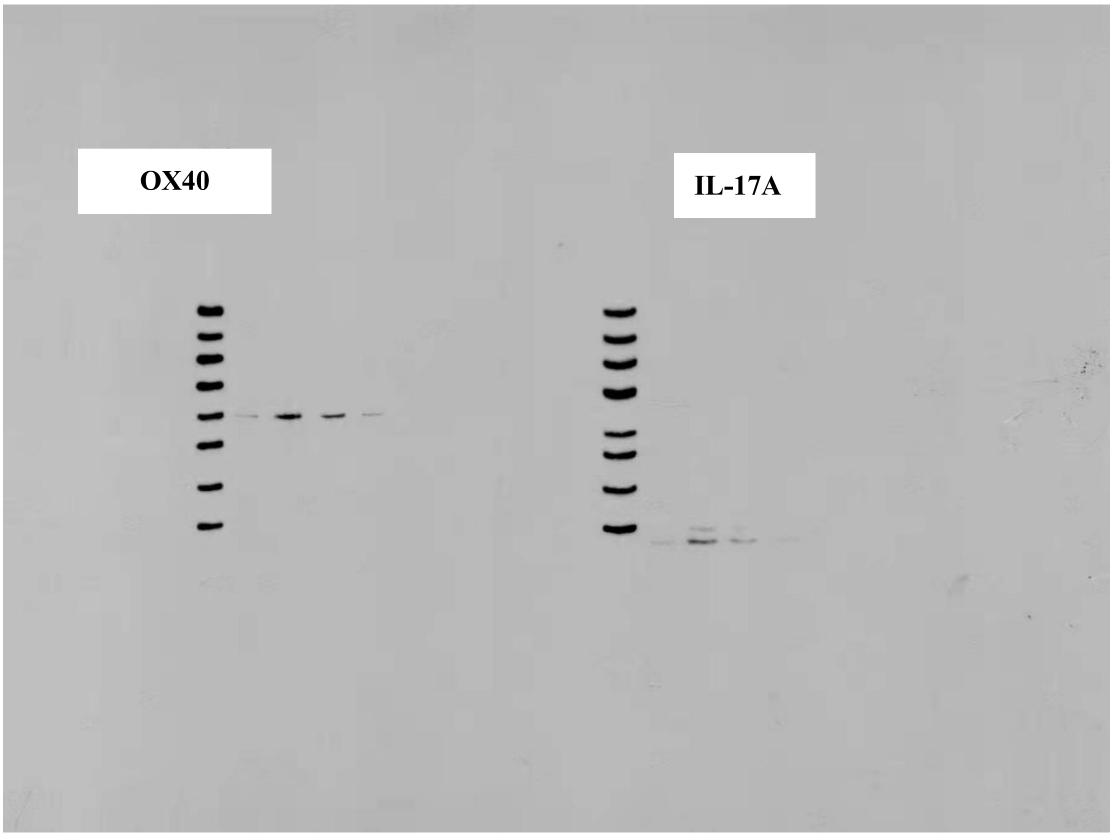

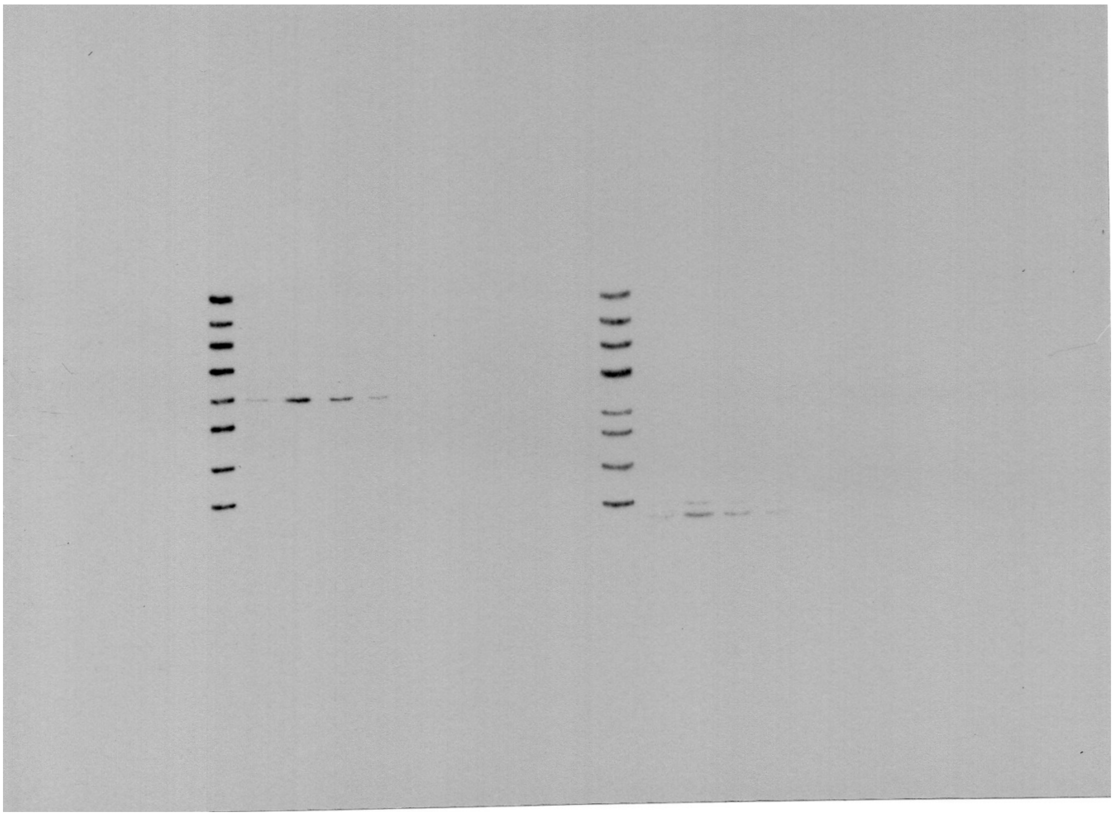

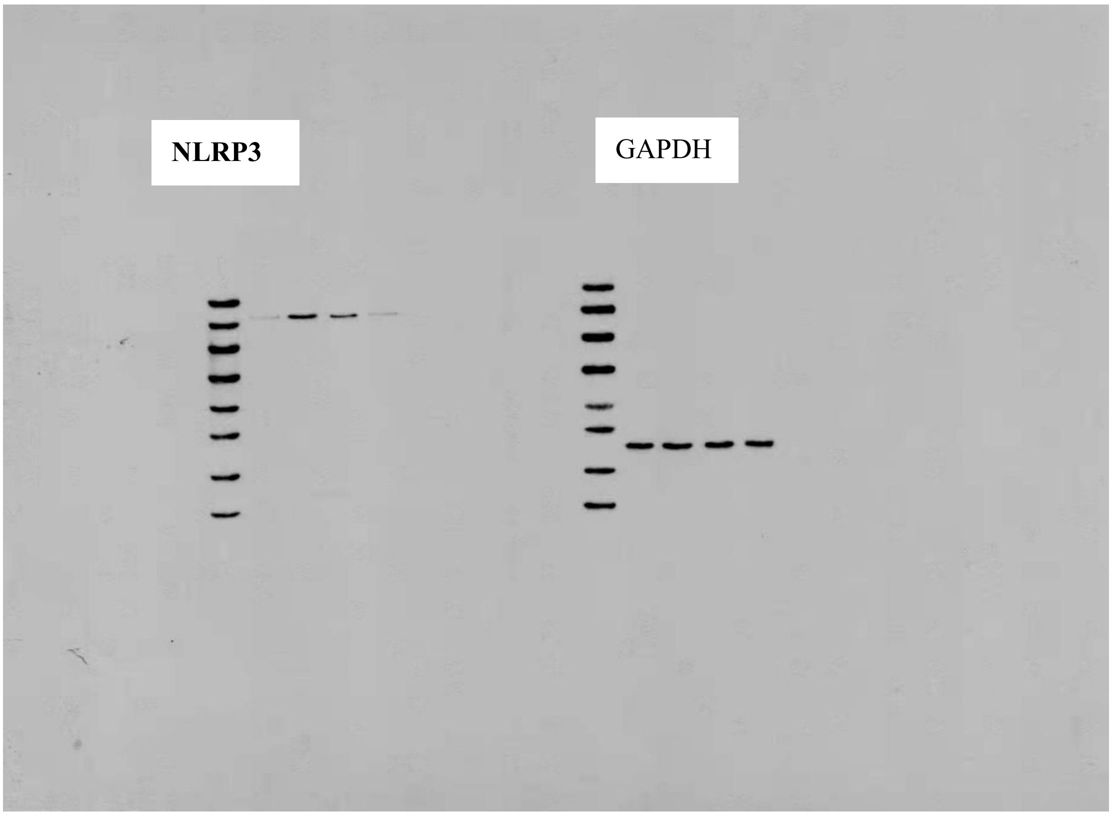
NLRP3
GAPDH

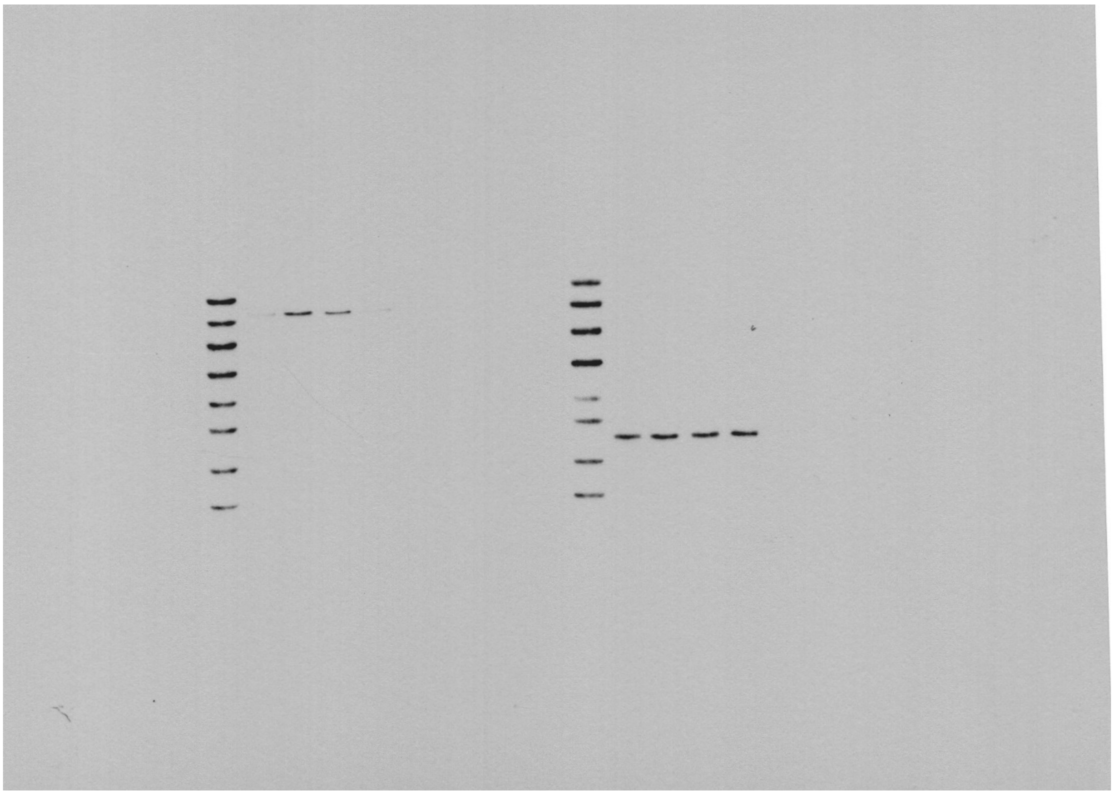

Figure S2. Representative western-blotting images of Nrf2, NQO1 in liver after arsenic exposure or *Lentinan* intervention

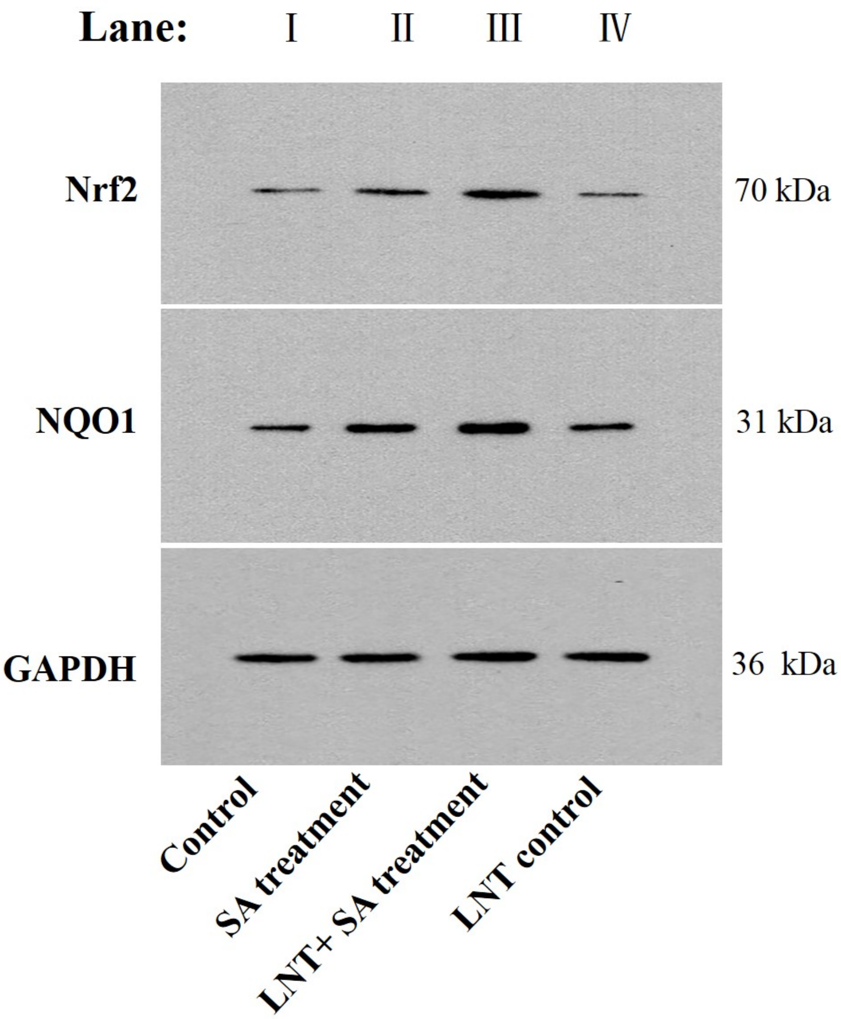

Notes: SA, sodium arsenite or $NaAsO_2$; LNT, *Lentinan*.

The original figure S2: Each figure has two exposures.

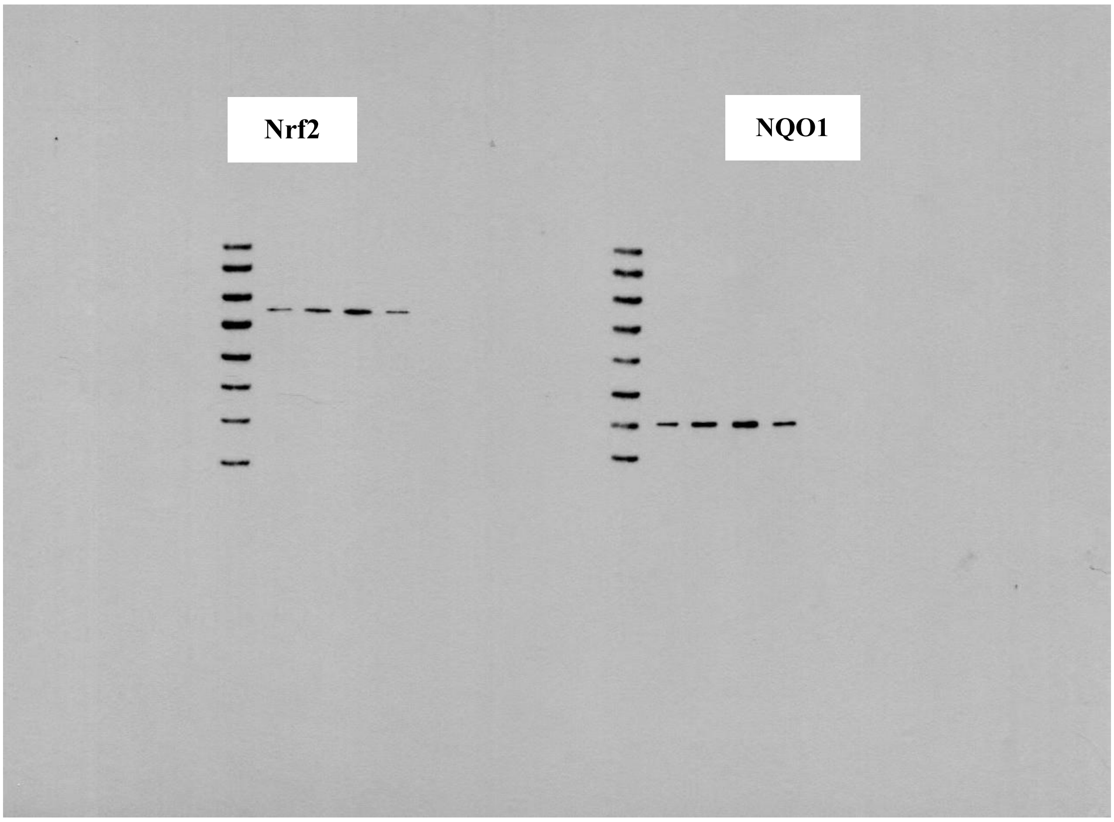

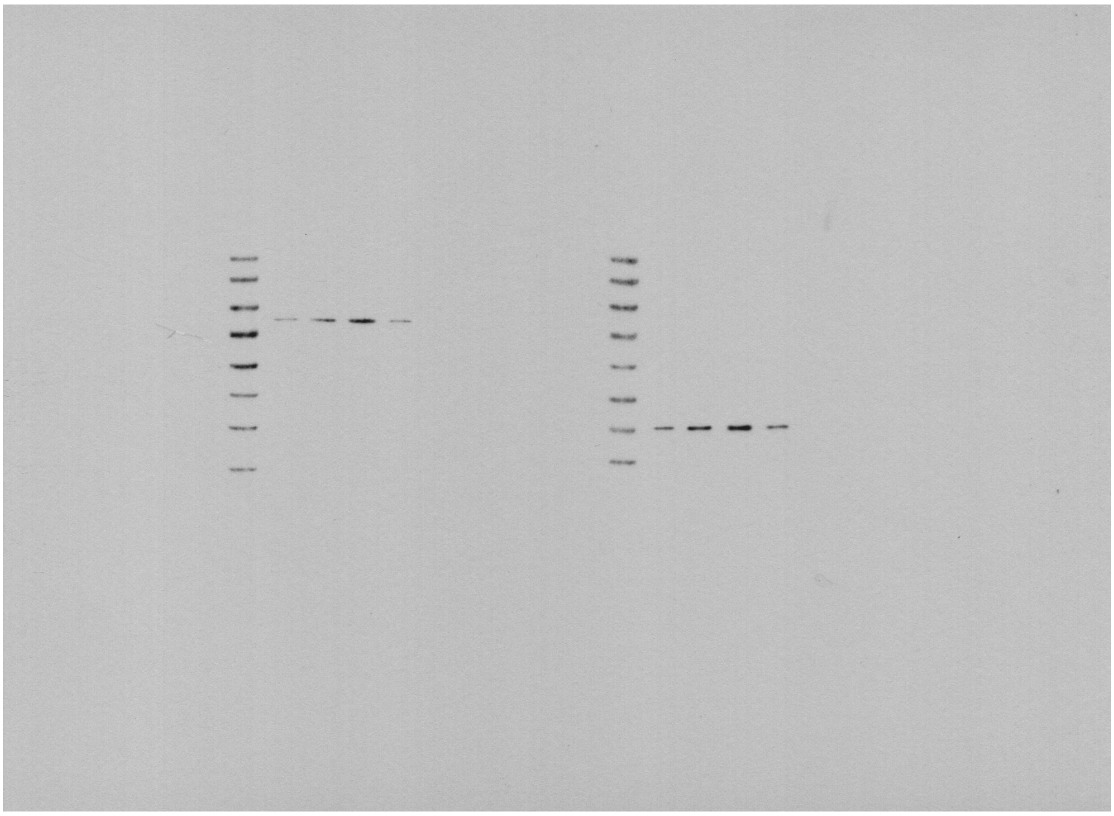

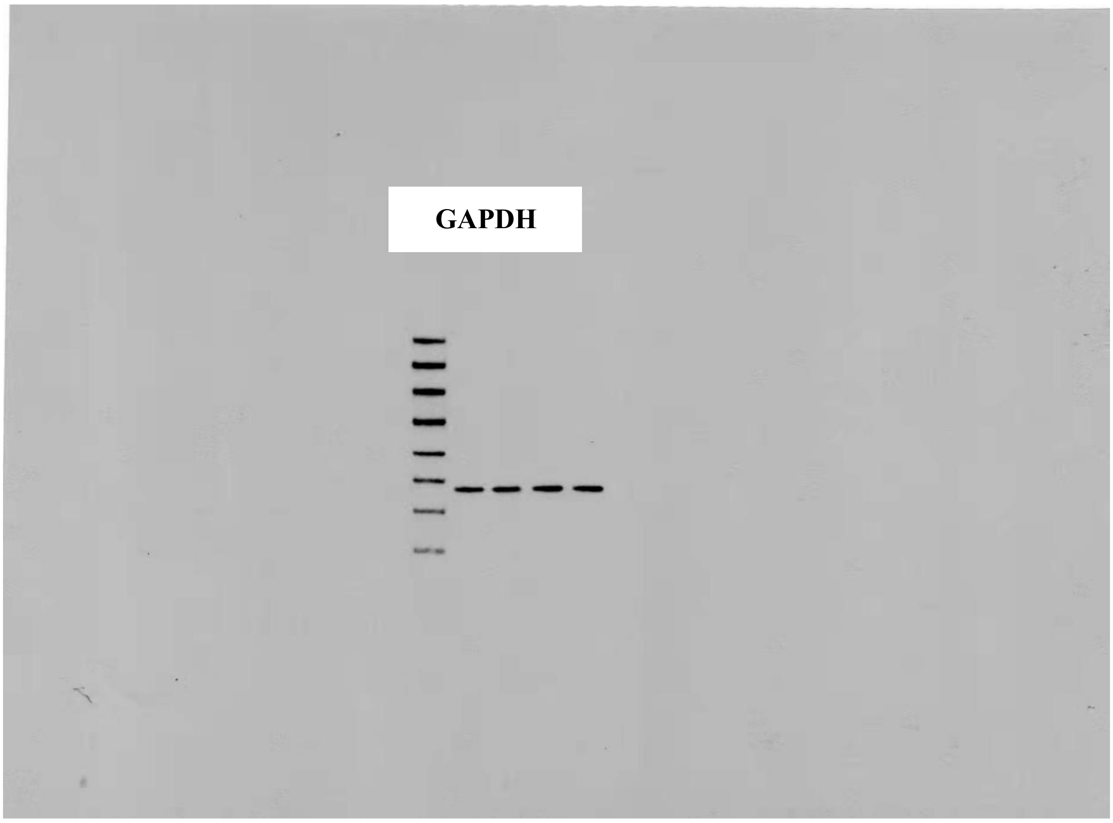
GAPDH

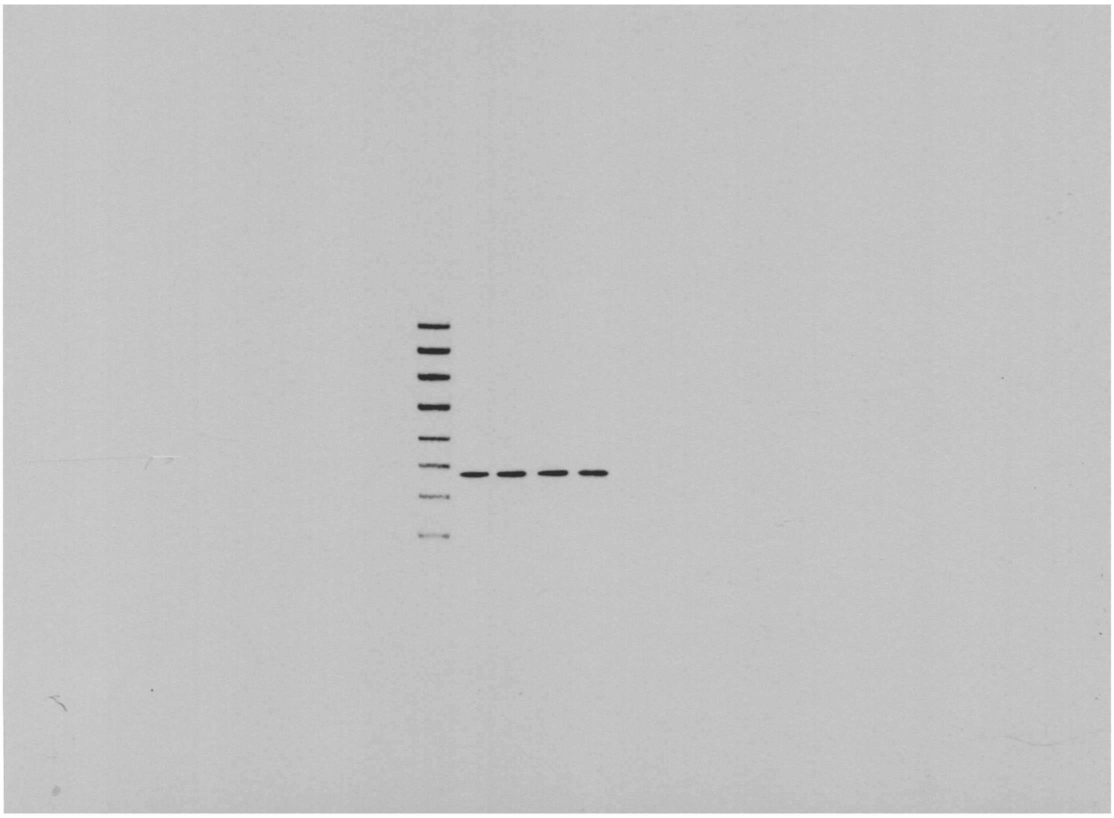

Supplemental Tables

Table S1. Characteristics of liver function in liver after arsenic exposure or LNT inTervention

Indices (units)	Control	SA treatment	LNT+SA treatment	LNT control
ALT (ng/ml)	5.47±0.49	8.78±0.76*	6.07±0.55$^{\#}$	5.75±0.43
AST (ng/ml)	7.14±0.57	12.42±1.87*	9.65±0.81$^{*\#}$	7.49±0.62

Notes: Dose of sodium arsenite (SA) was 10.0 mg/kg.bw by oral administration once every other day in the period of 14 days, and the dose of LNT intervention was 1.0 mg/kg.bw by intramuscular injection. Data were expressed as mean ± SD; n=5. Levels of ALT, AST in serum were quantified by the the convertion from OD values to ng values of per milliliter; Data were analyzed by one-way analysis of variance (ANOVA) and independent sample's t-test; $^{*}P<0.05$ indicates a significant difference compared with control group; $^{\#}P<0.05$ indicates a significant difference compared with SA treatment group.

Abbreviations: SA, sodium arsenite or $NaAsO_2$; LNT, Lentinan; ALT, alanine aminotransferase; AST, aspartate aminotransferase.

Table S2. Levels of Th17 and Tregs in liver after arsenic exposure or LNT inTervention

Indices (Unit)	Control	SA treatment	LNT+SA treatment	LNT control
Th17 (%, liver)	0.74±0.15	6.77±0.49*	4.82±0.54$^{*\#}$	0.48±0.18
Tregs (%, liver)	0.45+0.08	2.41+0.45*	2.56+0.47*	0.42+0.09

Notes: Data were expressed as mean ± SD; n=5. The units of Th17, Tregs were presented as percentage (%), which were analyzed by ANOVA and independent sample's t-test; $^{*}P<0.05$ indicates a significant difference compared with control; $^{\#}P<0.05$ indicates a significant difference compared with SA treatment.

Abbreviations: Th17, $CD4^{+}$ type 17 helper T cells; Tregs, $CD4^{+}CD25^{+}Foxp3^{+}$ regulatory T cells.

Table S3. Levels of oxidative stress, inflammation cytokines in liver after arsenic exposure or LNT inTervention

Indices (Unit)	Control	SA treatment	LNT+SA treatment	LNT control
GSH (nmol/g tissue)	730.94±30.81	593.40±23.25*	648.09±29.32*#	738.95±29.94
MDA (nmol/g tissue)	49.90±6.42	74.56±7.63*	56.64±6.59#	47.51±5.97
OX40 (pg/g tissue)	220.23+13.03	277.03+14.58*	251.06+13.68*#	226.18+12.81
IL-17A (nmol/g tissue)	118.53±7.92	162.48±10.75*	136.60±9.15*#	119.52±8.74

Notes: Data were expressed as mean ± SD; n=5. Significant difference was obtained by ANOVA test and independent sample's t-test. *P<0.05 indicates a significant difference compared with control; #P<0.05 indicates a significant difference compared with SA treatment.
Abbreviations: MDA, malondialdehyde; GSH, glutathione; OX40 (CD134), Tumor necrosis factor receptor superfamily member 4; IL, Interleukin.

Table S4. Western-blotting analysis of inflammatory or anti-oxidative signals in liver after arsenic exposure or LNT inTervention

Indices (IA/IA)	Control	SA treatment	LNT+SA treatment	LNT control
OX40	0.25±0.06	0.93±0.11*	0.64±0.08*#	0.22±0.07
IL-17A	0.09±0.04	0.87±0.10*	0.44±0.07*#	0.07±0.04
NLRP3	0.11±0.05	1.05±0.16*	0.76±0.09*#	0.10±0.05
Nrf2	0.33±0.07	1.09±0.13*	1.72±0.18*#	0.36±0.06
NQO1	0.62±0.14	1.24±0.15*	1.78±0.17*#	0.71±0.08

Notes: Data were expressed as mean ± SD; n=5. The levels of proteins expression were evaluated by the measurement of relative value of integrated absorbance (IA), quantified by the ratio of target protein vs. internal control in each group (IA/IA). The significant difference between groups was obtained by independent sample's t-test followed ANOVA. *P<0.05 indicates a significant

difference compared with control group; #P<0.05 indicates a significant difference compared with SA treatment group.

Abbreviations: NLRP3, NOD-like receptor family pyrin domain-containing 3; Nrf2, NF-E2 p45-related factor 2; NQO1, NAD(P)H quinone dehydrogenase 1.
