# Supplementary material for: Lentinan alleviates arsenic-induced hepatotoxicity in mice via downregulation of OX40/IL-17A and activation of Nrf2 signaling
Source: BMC Pharmacol Toxicol. 2022 Mar 22;23:16. doi: 10.1186/s40360-022-00557-7 (PMC8939159; doi:10.1186/s40360-022-00557-7)

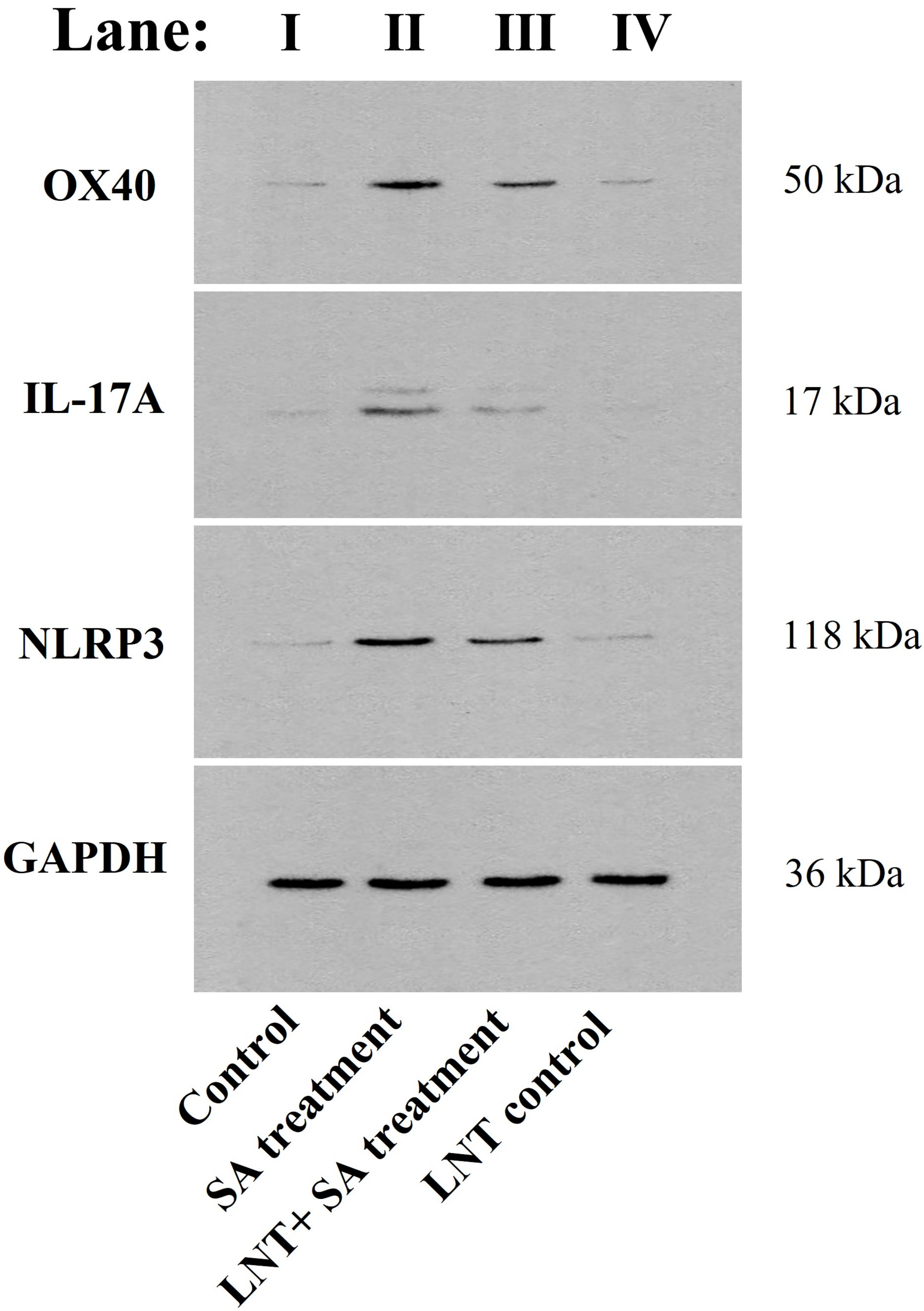
Lane: I II III IV
OX40
50 kDa
IL-17A
17 kDa
NLRP3
118 kDa
GAPDH
36 kDa
Control
SA treatment
LNT+ SA treatment
LNT control

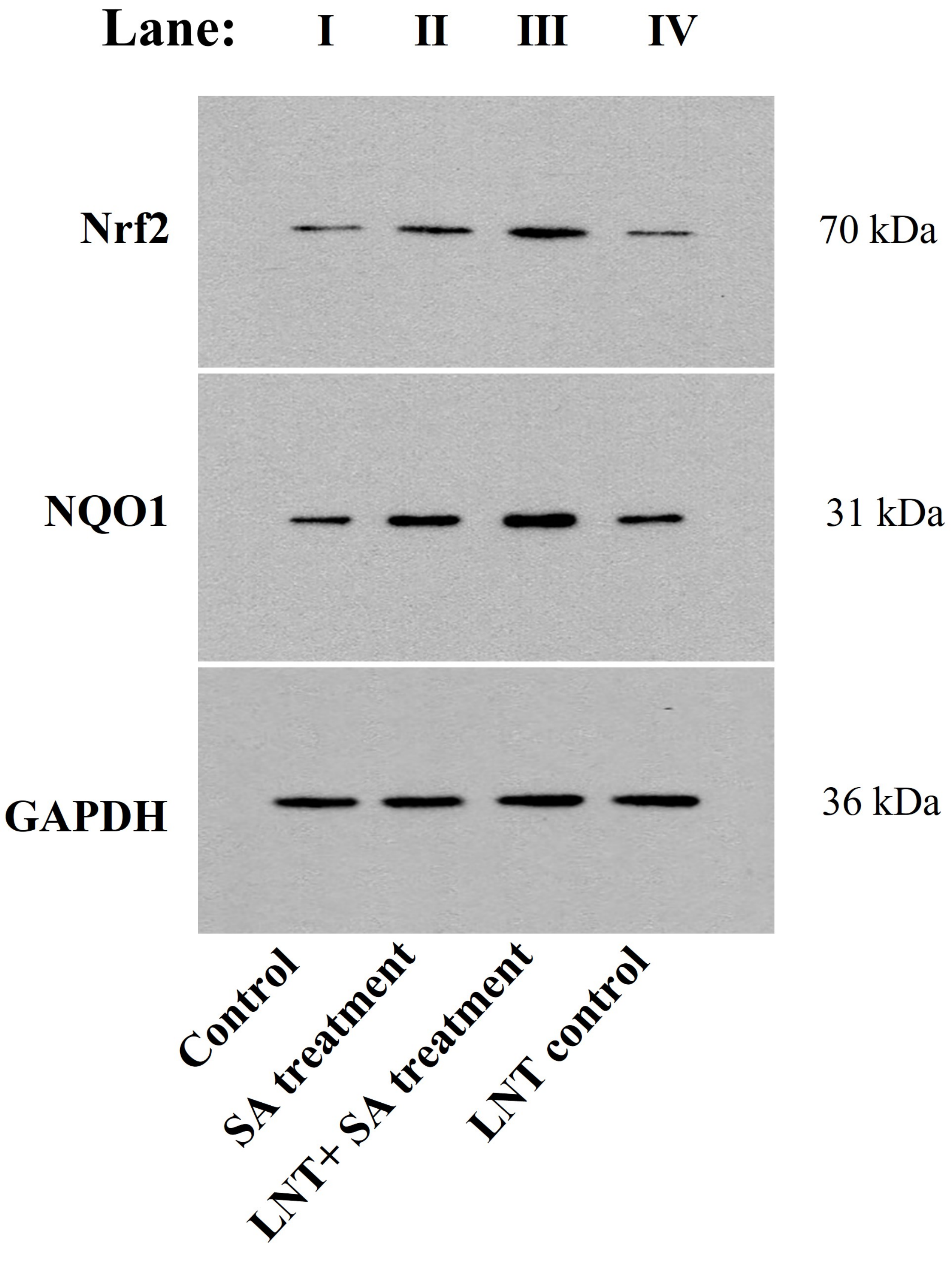
Lane:
I
II
III
IV
Nrf2
70 kDa
NQO1
31 kDa
GAPDH
36 kDa
Control
SA treatment
LNT+ SA treatment
LNT control

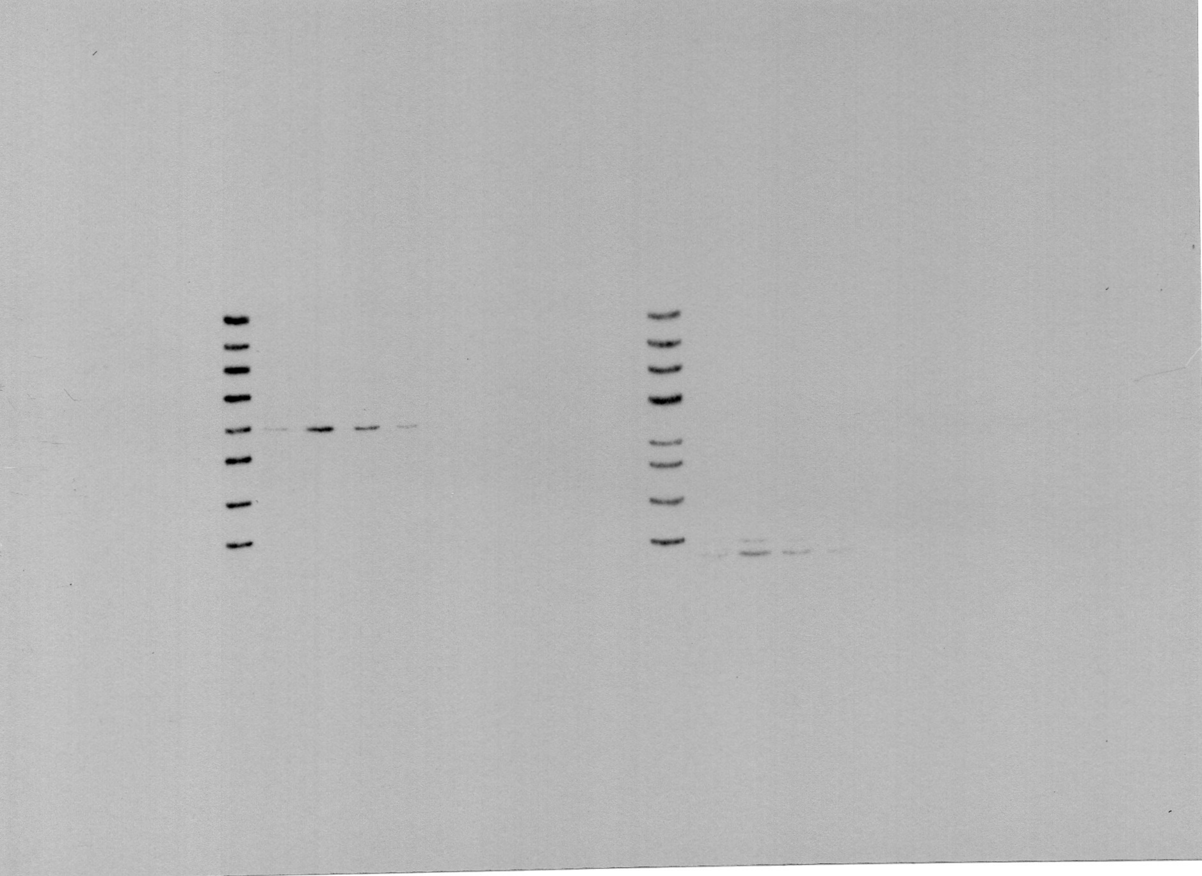

OX40: 50 kDa

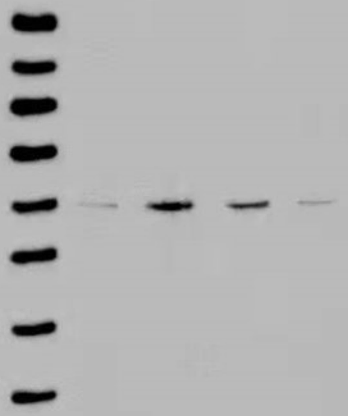

IL-17A: 17 kDa

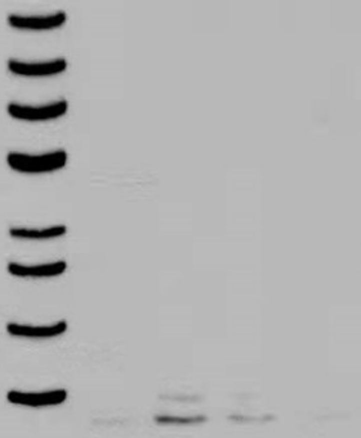

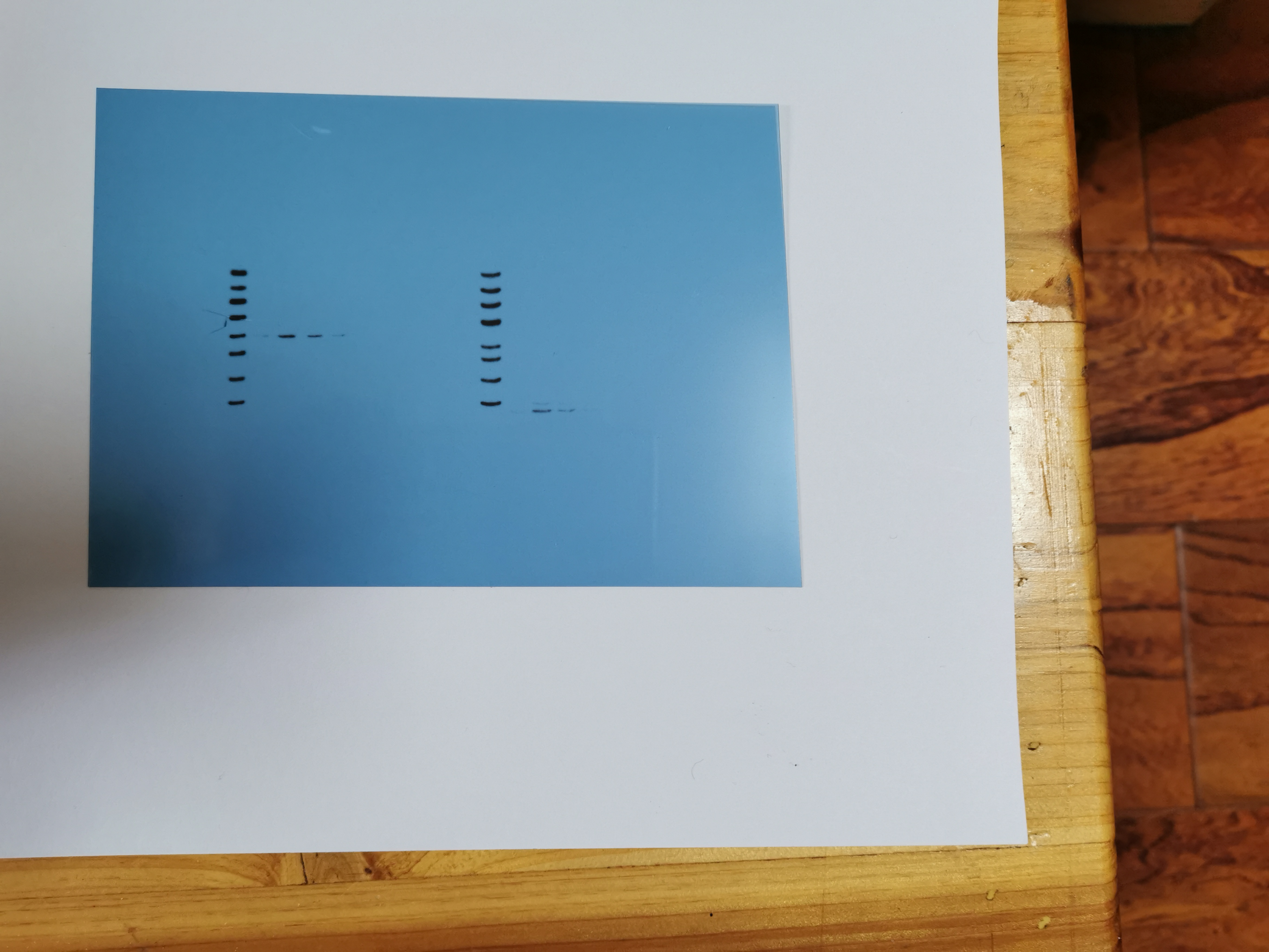

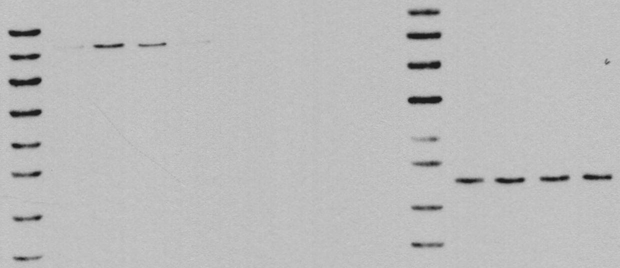

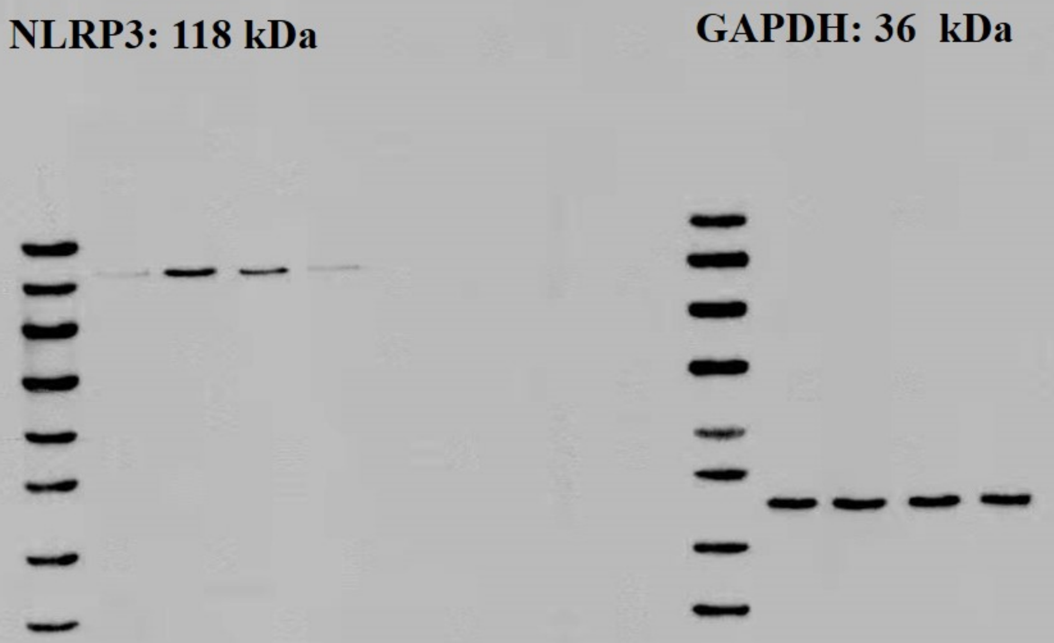
NLRP3: 118 kDa
GAPDH: 36 kDa

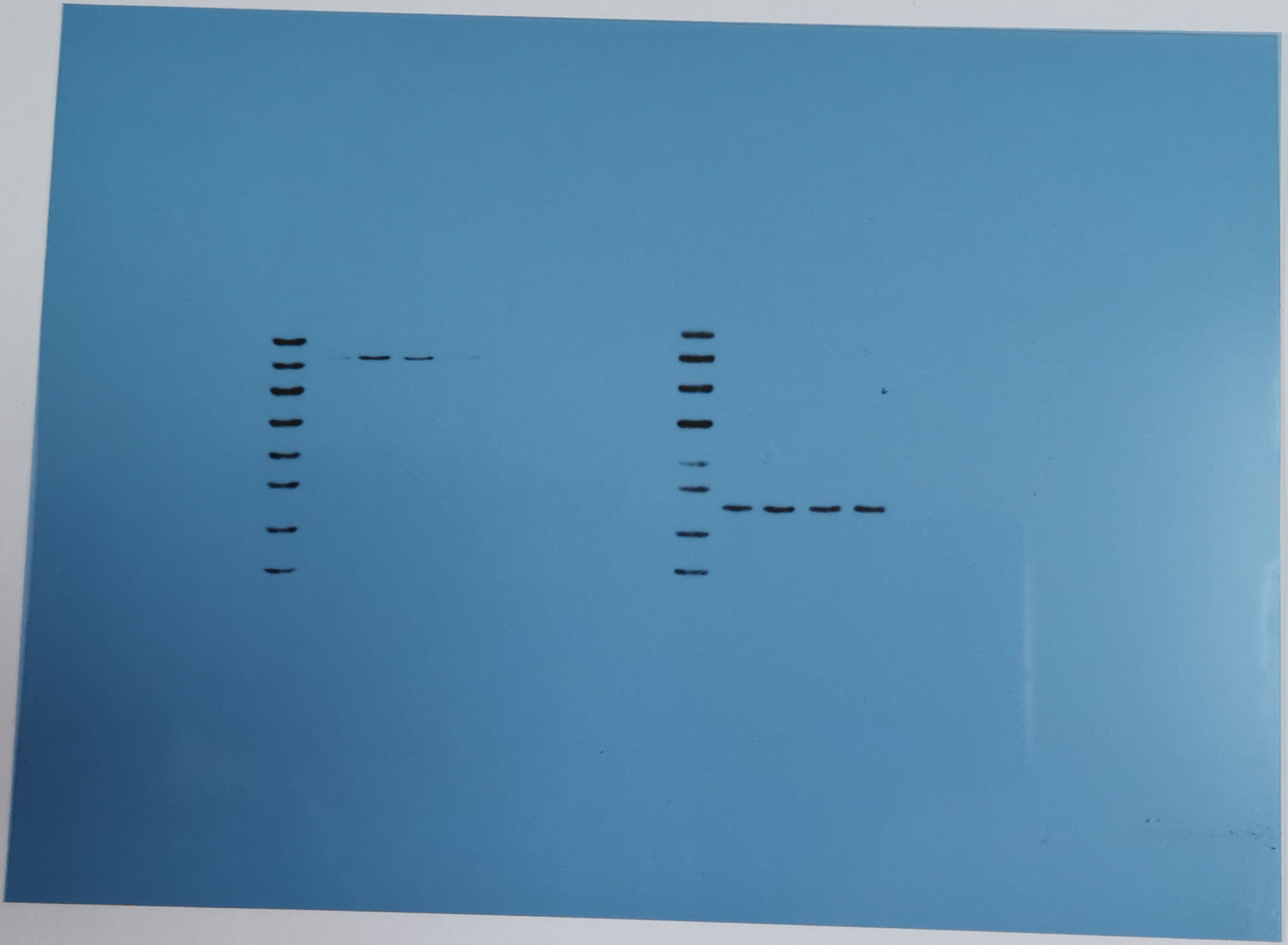

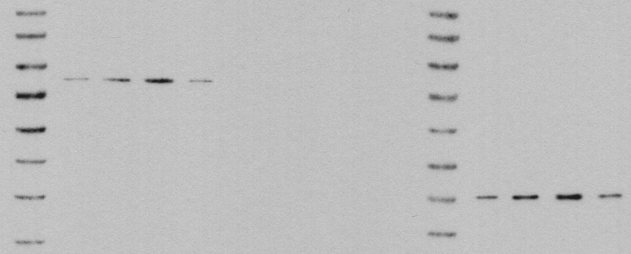

Nrf2: 70 kDa

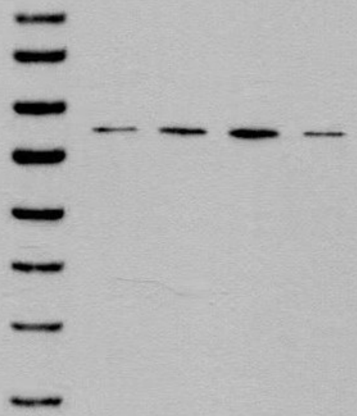

NQO1: 31 kDa

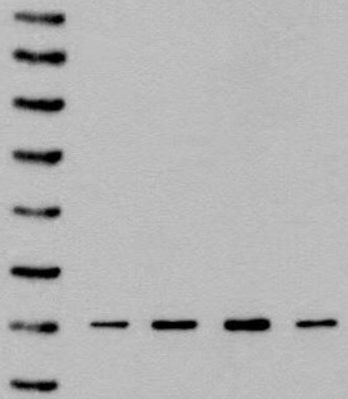

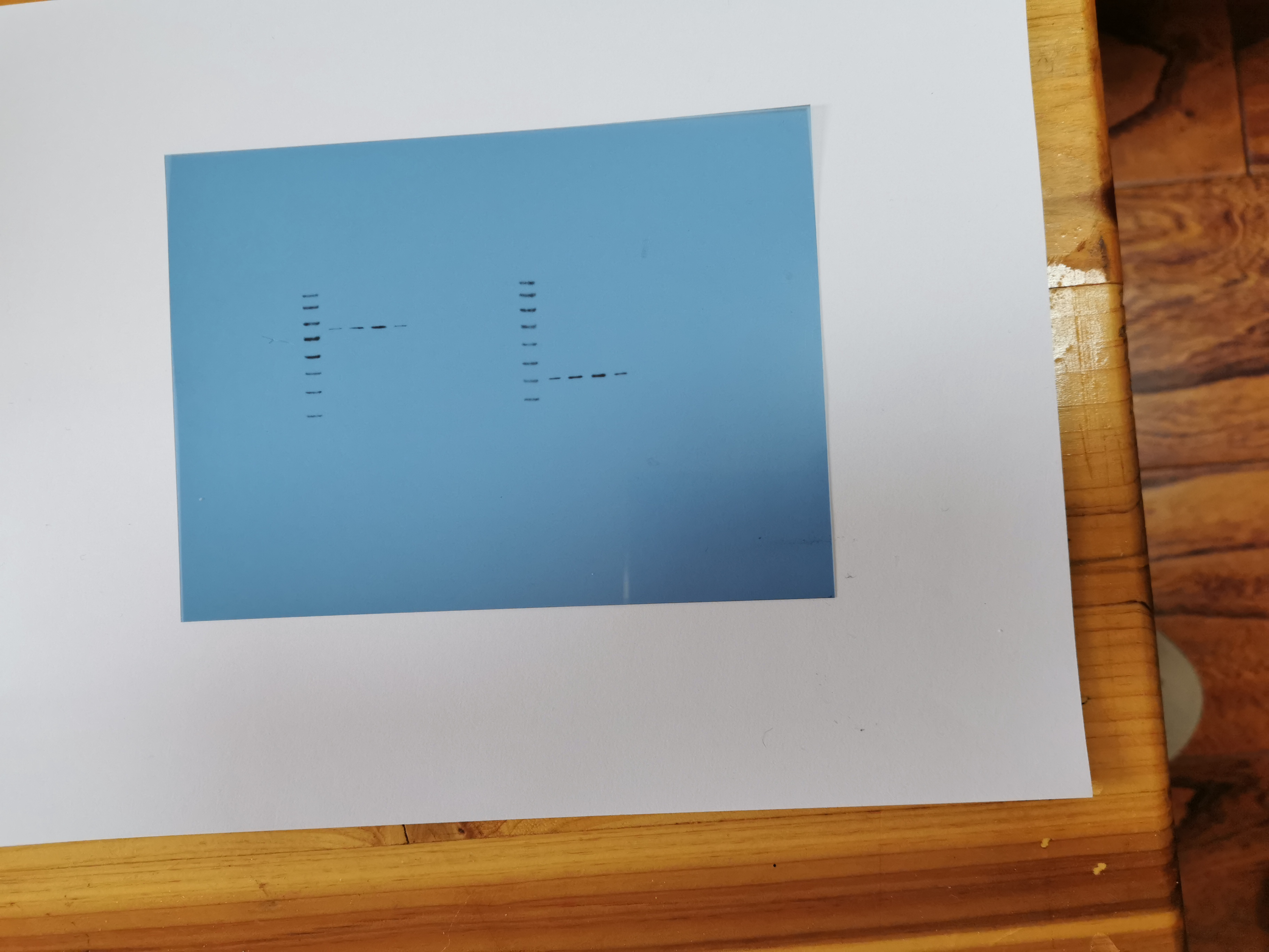

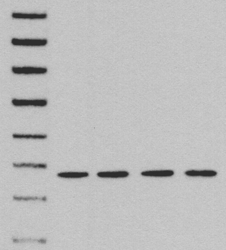

GAPDH: 36 kDa

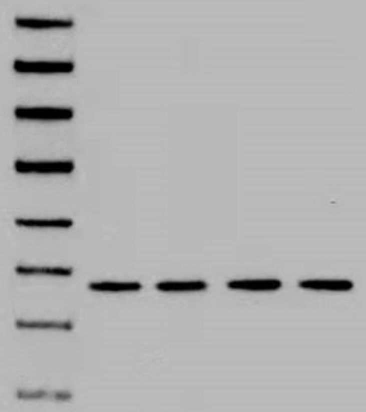

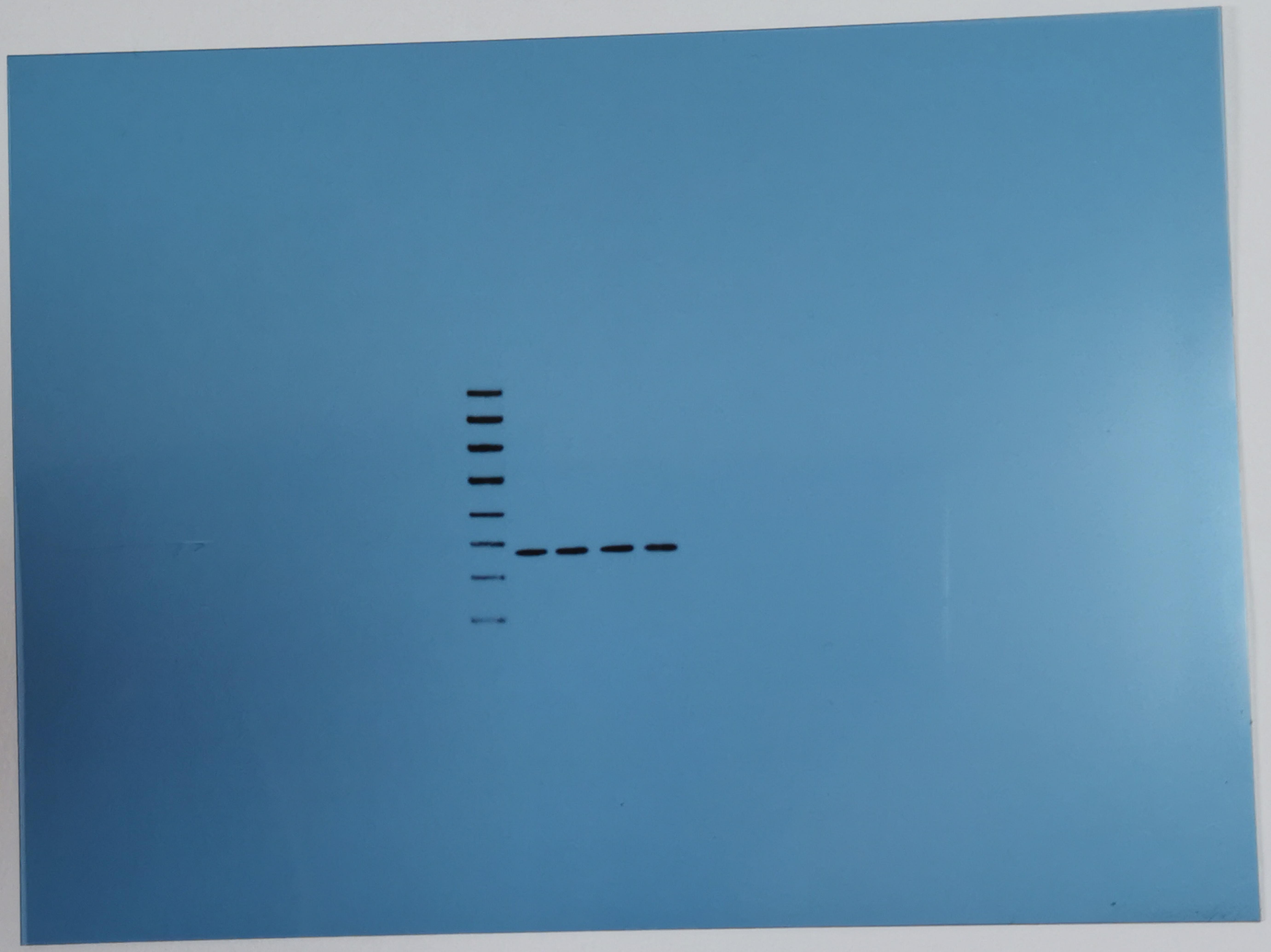

Supplement: Supplementary file 1 — Additional file 1. [file 40360_2022_557_MOESM1_ESM.zip › Supplemental Figures & The Original Figures.pdf]
